# Safety and immunogenicity of the RTS,S/AS01 malaria vaccine in infants and children identified as HIV-infected during a randomized trial in sub-Saharan Africa

**DOI:** 10.1016/j.vaccine.2019.10.077

**Published:** 2019-11-07

**Authors:** Lucas Otieno, Yolanda Guerra Mendoza, Samuel Adjei, Tsiri Agbenyega, Selidji Todagbe Agnandji, Pedro Aide, Pauline Akoo, Daniel Ansong, Kwaku Poku Asante, James A Berkley, Samwel Gesase, Mary J. Hamel, Irving Hoffman, Seyram Kaali, Portia Kamthunzi, Simon Kariuki, Peter Kremsner, Miguel Lanaspa, Bertrand Lell, Marc Lievens, John Lusingu, Anangisye Malabeja, Nahya Salim Masoud, Ali Takadir Mtoro, Patricia Njuguna, Opokua Ofori-Anyinam, Godfrey Allan Otieno, Walter Otieno, Seth Owusu-Agyei, Lode Schuerman, Hermann Sorgho, Marcel Tanner, Halidou Tinto, Innocent Valea, Pascale Vandoolaeghe, Jahit Sacarlal, Martina Oneko

**Affiliations:** a KEMRI-Walter Reed Project, Kombewa, Kenya; b GSK, Wavre, Belgium; c Kwame Nkrumah University of Science and Technology, Kumasi, Ghana; d Centre de Recherches Médicales de Lambaréné, Lambaréné, Gabon and Institute of Tropical Medicine, University of Tübingen, Tübingen, Germany; e Centro de Investigação em Saúde de Manhiça, Manhiça, Mozambique; f National Institute of Health, Ministry of Health, Maputo, Mozambique; g Kenya Medical Research Institute-Wellcome Trust Research Programme, Centre for Geographic Medicine Research, Kilifi, Kenya; h Kintampo Health Research Center, Kintampo, Ghana; i Centre for Tropical Medicine & Global Health, Nuffield Department of Medicine, University of Oxford, Oxford, United Kingdom; j National Institute for Medical Research, Korogwe, Tanzania; k Malaria Branch Division of Parasitic Diseases and Malaria, Center for Global Health, Centers for Disease Control and Prevention, Atlanta, GA, USA; l Department of Medicine, University of North Carolina, Chapel Hill, NC, USA; m Kenya Medical Research Institute, Centre for Global Health Research, Kisumu, Kenya; n Barcelona Institute for Global Health (ISGlobal), Hospital Clínic-Universitat de Barcelona, Spain; o Muhimbili University of Health and Allied Sciences (MUHAS), Dar es Salaam, Tanzania; p Ifakara Health Institute, Bagamoyo, Tanzania; q Pwani University, Kilifi, Kenya; r Diseases Control Department, London School of Hygiene and Tropical Medicine, London, UK; s Institut de Recherche en Sciences de la Santé, Nanoro, Burkina Faso; t Swiss Tropical and Public Health Institute, Basel, Switzerland and University of Basel, Basel, Switzerland; u Faculdade de Medicina, Universidade Eduardo Mondlane (UEM), Maputo, Mozambique

**Keywords:** HIV, Malaria, RTS, S/AS01 vaccine, Safety, Immunogenicity, Children

## Abstract

**Background:**

We assessed the safety and immunogenicity of the RTS,S/AS01 malaria vaccine in a subset of children identified as HIV-infected during a large phase III randomized controlled trial conducted in seven sub-Saharan African countries.

**Methods:**

Infants 6–12 weeks and children 5–17 months old were randomized to receive 4 RTS,S/AS01 doses (R3R group), 3 RTS,S/AS01 doses plus 1 comparator vaccine dose (R3C group), or 4 comparator vaccine doses (C3C group) at study months 0, 1, 2 and 20. Infants and children with WHO stage III/IV HIV disease were excluded but HIV testing was not routinely performed on all participants; our analyses included children identified as HIV-infected based on medical history or clinical suspicion and confirmed by polymerase chain reaction or antibody testing. Serious adverse events (SAEs) and anti-circumsporozoite (CS) antibodies were assessed.

**Results:**

Of 15459 children enrolled in the trial, at least 1953 were tested for HIV and 153 were confirmed as HIV-infected (R3R: 51; R3C: 54; C3C: 48). Among these children, SAEs were reported for 92.2% (95% CI: 81.1–97.8) in the R3R, 85.2% (72.9–93.4) in the R3C and 87.5% (74.8–95.3) in the C3C group over a median follow-up of 39.3, 39.4 and 38.3 months, respectively. Fifteen HIV-infected participants in each group (R3R: 29.4%, R3C: 27.8%, C3C: 31.3%) died during the study. No deaths were considered vaccination-related. In a matched case-control analysis, 1 month post dose 3 anti-CS geometric mean antibody concentrations were 193.3 EU/mL in RTS,S/AS01-vaccinated HIV-infected children and 491.5 EU/mL in RTS,S/ AS01-vaccinated immunogenicity controls with unknown or negative HIV status (p = 0.0001).

**Conclusions:**

The safety profile of RTS,S/AS01 in HIV-infected children was comparable to that of the comparator (meningococcal or rabies) vaccines. RTS,S/AS01 was immunogenic in HIV-infected children but antibody concentrations were lower than in children with an unknown or negative HIV status.

## 1 Introduction

Malaria and human immunodeficiency virus (HIV) infection remain among the most important public health challenges of our times. An estimated 219 million malaria cases and 435,000 malaria-related deaths occurred worldwide in 2017, with sub-Saharan Africa carrying more than 90% of the global malaria burden [1]. Of all malaria-related deaths worldwide, 61% were in children under 5 years old [1]. In 2017, of the estimated 36.9 million people living with HIV and 940,000 HIV-related deaths globally, approximately 70% were in sub-Saharan Africa [2]. Children younger than 15 years account for approximately 5% of all people living with HIV and 12% of all HIV-related deaths [2]. Most of these children are infected with HIV *in utero*, during birth or through breastfeeding.

Given their geographic overlap, malaria and HIV co-infection is common in sub-Saharan Africa [3,4]. It is therefore important to evaluate the safety and immunogenicity of any malaria candidate vaccine intended for use in this region in HIV-infected children. The pre-erythrocytic RTS,S/AS01 vaccine (GSK) contains portions of the *Plasmodium falciparum* circumsporozoite (CS) protein and has been shown to be effective in reducing the malaria burden in children when used alongside other malaria interventions in phase II and III clinical trials [5–10]. The safety profile of the vaccine is in line with that of other pediatric vaccines [5–10] and RTS,S/AS01 received a positive scientific opinion from the European Medicines Agency in 2015 [11]. The World Health Organization (WHO) recommended pilot implementation of the vaccine in children 5– 17 months of age in sub-Saharan Africa to answer outstanding questions on feasibility of reaching children with the 4 doses needed for optimal benefit, monitor the safety profile of RTS,S/ AS01 in real-life settings, and measure impact [12].

One prior randomized controlled phase III trial specifically assessed the safety and immunogenicity of a 3-dose RTS,S/AS01 vaccination series in Kenyan children with WHO stage I or II HIV disease, most of whom received antiretroviral therapy (ART) to delay disease progression and cotrimoxazole prophylaxis to prevent opportunistic infections. This prior trial showed that RTS,S/ AS01 was well tolerated and immunogenic in HIV-infected children on ART [13].

Here, we report results of RTS,S/AS01 vaccination in a small subset of children identified as HIV-infected during a separate large phase III randomized controlled efficacy trial which enrolled more than 15,000 children in seven countries in sub-Saharan Africa. The efficacy, safety and immunogenicity findings for the overall population enrolled in this trial, comparing a 3- or 4-dose RTS,S/AS01 schedule with 4 doses of comparator vaccines, were published [7–10]. Although HIV testing was not routinely performed, a small proportion of participants were identified as HIV-infected during the trial based on their medical condition, with HIV diagnoses made in the context of the routine clinical care provided at the study centers (and confirmation by polymerase chain reaction [PCR] or antibody testing). This subset therefore provided another opportunity to evaluate safety and immunogenicity of RTS,S/AS01 in HIV-infected children.

## 2 Methods

### 2.1 Study design, participants and vaccines

The initial large phase III double-blind (observer-blind) randomized controlled efficacy trial was performed between 27 March 2009 and 31 January 2014 at eleven centers in seven countries in sub-Saharan Africa: Burkina Faso, Gabon, Ghana, Kenya, Malawi, Mozambique and Tanzania. The study centers represented different geographic areas with diverse malaria transmission intensities [9,14] and overlapping background HIV prevalence [2]. Details on the study centers and methods have been described previously [7–10,15–18]. Insecticide-treated bednets were made available to all screened children, and bednet use was high across study groups (approximately 85% in participants enrolled at 6–12 weeks of age and 75% in participants enrolled at 5–17 months of age) but varied between study centers [10].

The study was registered on ClinicalTrials.gov (NCT00866619) and was conducted in accordance with the Declaration of Helsinki, International Conference on Harmonization Good Clinical Practice guidelines and local rules and regulations. The protocol and other study-related documents were approved by the ethical review board at each study center and partner institutions and by the national regulatory authority in each country. A summary of the protocol is available at https://www.gsk-studyregister.com/study/3251. Anonymized individual participant data and study documents can be requested for further research from www.clinicalstudydatarequest.com. All participants’ parents or legally authorized representatives (LARs) provided signed or thumb-printed and witnessed informed consent prior to enrollment. An independent data monitoring committee oversaw the safety of the participants by regular review of unblinded study data [10].

In the initial trial, we enrolled infants 6–12 weeks of age and children 5–17 months of age (irrespective of their HIV status) and randomized them 1:1:1 to receive either 4 doses of RTS,S/ AS01 vaccine (R3R group), 3 doses of RTS,S/AS01 vaccine followed by a dose of comparator vaccine (R3C group), or 4 doses of comparator vaccine (C3C group). Vaccines in the three groups were given intramuscularly in the thigh or deltoid (depending on the participant’s age) at study months 0, 1, 2 and 20. RTS,S/AS01 contains portions of the *P. falciparum* CS protein, fused to hepatitis B surface antigen (HBsAg), together with free HBsAg and is adjuvanted with the adjuvant system (AS) AS01 [19]. For the first 3 doses, we used a meningococcal C CRM197 conjugate vaccine (MenC-CRM, *Menjugate*, Novartis) as comparator in infants enrolled at 6–12 weeks of age and a rabies vaccine *(Verorab*, Sanofi Pasteur) in children enrolled at 5–17 months of age. For the fourth dose, we used MenC-CRM as comparator in both age categories. Infants in the 6–12 weeks age category received other routine pediatric vaccines according to local Expanded Program on Immunization policy [10].

The children and their parents/LARs as well as those responsible for the evaluation of study endpoints were unaware of which vaccine was given. The study staff responsible for preparation and administration of vaccines (and thus aware of vaccine assignment) were not involved in any of the analyses.

Detailed inclusion and exclusion criteria for enrollment in the initial study have been described previously [10]. Children with active WHO stage III or IV HIV disease [20] at screening were excluded from the initial study. The current analyses included children identified as HIV-infected, based on their general medical history taken at screening or based on clinical suspicion during the course of the trial, and confirmed to be HIV positive by PCR or— for children 18 months or older—by antibody testing. Importantly, HIV testing was not a trial procedure and was thus not routinely performed on all participants. HIV-testing practices differed between centers, depending on national guidelines and local practices. Consequently, some HIV-infected children might not have been identified as such. Voluntary counseling and testing, highly active ART and prevention of mother-to-child transmission were available at the study centers or in health facilities within the study area. The centers followed the national recommendations for the treatment and management of HIV-infected children. Data on ART use were not systematically collected during the study.

We also performed a matched case-control analysis to compare RTS,S/AS01 immunogenicity in children confirmed as HIV-infected with that in controls not identified as HIV-infected. This immunogenicity control cohort included children with unknown HIV status (not tested) and children tested and found to be HIV-negative.

### 2.2 Safety outcomes and assessments

We assessed the occurrence of serious adverse events (SAEs) in HIV-infected children, within one month following each vaccine dose and over the entire follow-up period. In the protocol, these analyses were planned by age category, but because of the limited number of confirmed HIV-infected children, they were also performed pooled across age categories. SAEs were defined as any untoward medical occurrence that resulted in death, was life-threatening, required hospitalization or prolongation of existing hospitalization or resulted in disability/incapacity. SAEs were collected by passive surveillance at the health facilities and through monthly home visits. Seizures occurring within 30 days of vaccination were also collected as SAEs; those occurring within 7 days post-vaccination were further analyzed according to the Brighton collaboration guidelines [21]. Potential immune-mediated disorders (pIMDs) occurring throughout the study were collected as SAEs according to a predefined list provided in the study protocol [22]. HIV infection could be reported as an SAE during the course of the study based on the investigators’ judgment of whether it met the criteria of an SAE. To enable accurate SAE reporting, investigators were encouraged to perform HIV testing if HIV infection was suspected. We also assessed the occurrence of fatal SAEs in boys and girls separately (*post hoc* analysis).

The investigators assessed the relationship between the reported SAEs and vaccination based on their clinical judgement. Verbal autopsies were performed on deaths that occurred outside the health facilities according to WHO guidelines [23]. The cause of death was determined by a panel of three experienced verbal autopsy reviewers, as detailed previously [22].

### 2.3 Immunogenicity outcomes and assessments

We assessed anti-CS antibody concentrations on blood samples collected at screening and 1 month post-dose 3 in HIV-infected children. Analyses in the HIV-infected population were done for each age category (as planned per protocol) and pooled across age categories. The analyses of anti-CS antibody responses at later time points (18 and 30 months post-dose 3), planned per protocol, were canceled because HIV-infected participants who survived until these later time points may differ from those who did not (e.g., by having less severe HIV illness or by accessing ART) and including them could potentially introduce bias related to the anti-CS antibody response. The analyses of anti-HBs antibody responses were also canceled because anti-HBs results had mean-while been published for the prior study that was specifically designed to assess RTS,S/AS01 immunogenicity in HIV-infected children [13]. Anti-CS antibodies were measured at the Center for Vaccinology (Ghent University, Belgium) using an enzyme-linked immunosorbent assay (ELISA) based on the binding of serum antibodies to the recombinant R32LR protein, as described previously [24]. The assay cut-off was 0.5 ELISA units (EU)/mL.

### 2.4 Statistical analyses

No formal sample size calculations were performed for the analyses on the HIV-infected subset since this subset was defined by the total population enrolled in the initial trial [7–9] and the number of participants confirmed as HIV-infected. Statistical analyses of the endpoints presented here were descriptive and were performed using SAS Drug Development.

We performed all safety assessments on the intent-to-treat (ITT) population of HIV-infected participants, which included all HIV-infected children confirmed throughout the study (as described above) who had received at least 1 dose of RTS,S/AS01 or comparator vaccine. Safety assessments pertaining to the 30-day period after the fourth dose were performed on HIV-infected participants who had received a fourth dose. We calculated the proportions of participants reporting SAEs, classified by Medical Dictionary for Regulatory Activities preferred term, with exact 95% confidence intervals (CIs) for the various follow-up periods. We generated survival curves based on all follow-up data from dose 1 to study end for the individual and pooled age categories. A log-rank test was performed on the three groups.

We performed the primary immunogenicity assessments on the per protocol population which included all participants confirmed HIV-infected by 2 May 2012 (data lock point when most children had performed their study month 20 visit), who were primed with 3 doses of RTS,S/AS01 or comparator vaccine per protocol and complied with all protocol-defined procedures. We calculated the percentages of participants who were seropositive for anti-CS antibodies (i.e., with concentrations ≥0.5 EU/mL) and anti-CS antibody geometric mean concentrations (GMCs) with 95% CIs.

The matched case-control analysis was performed on the total vaccinated population of HIV-infected cases and immunogenicity controls. Each participant confirmed as HIV-infected by the data lock point, primed with 3 doses of RTS,S/AS01 and with an anti-CS serology result available at 1 month post-dose 3 (case), was matched (1:1) with another RTS,S/AS01-vaccinated child not identified as HIV-infected by the data lock point (immunogenicity control with HIV-negative or unknown HIV status). The matching algorithm identified all children who had received the first 3 doses of RTS,S/AS01, who were not confirmed as HIV-infected, had an anti-CS result available for the 1 month post-dose 3 timepoint, were in the same treatment group as the HIV-infected child, had identical values on all considered matching variables (center, sex, age category, ethnic group, and the number of hepatitis B vaccine doses received prior to RTS,S/AS01 dose 1), and were not yet selected as immunogenicity control for another HIV-infected child. If several participants were eligible as immunogenicity controls for a certain HIV-infected child, the control was randomly selected from these participants. To assess if the anti-CS response differed between HIV-infected participants and immunogenicity controls, we compared the log-transformed mean anti-CS concentrations between both groups using a paired *t*-test.

## 3 Results

### 3.1 Study population

**Fig. 1:**
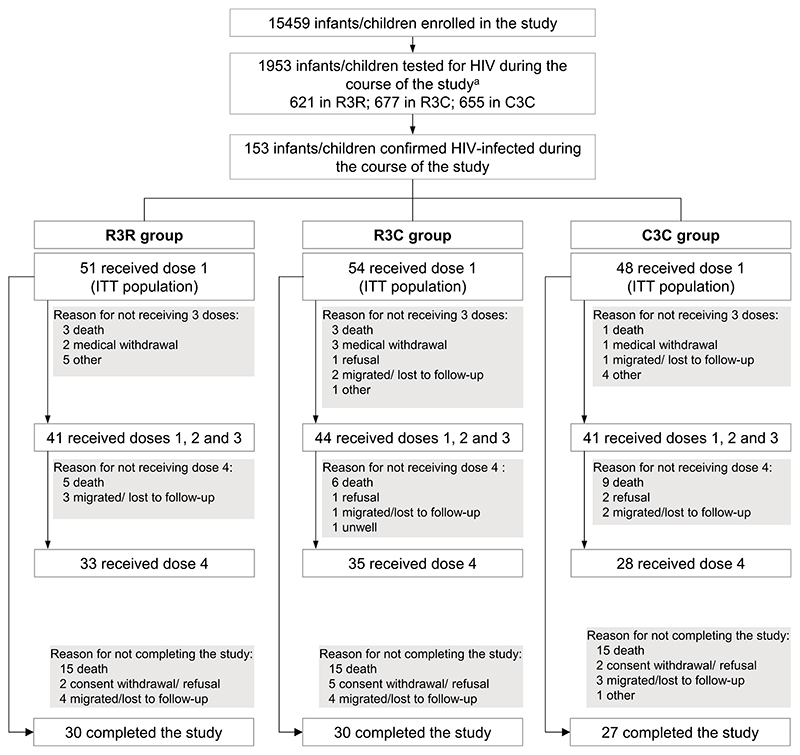
Flow diagram. R3R, group receiving 4 doses of RTS,S/AS01; R3C, group receiving 3 doses of RTS,S/AS01 plus 1 dose of comparator vaccine; C3C, group receiving 4 doses of comparator vaccine; ITT, intent-to-treat. ^a^Not all HIV tests were recorded in the database for all centers; 1953 is therefore the minimum number of children tested for HIV at least once during the study.

15,459 participants were enrolled in the initial large phase III trial (8922 in the 5-17 months and 6537 in the 6-12 weeks age category), 1953 were reported to have been tested for HIV at least once and 153 were confirmed as HIV-infected during the study: 51/5156 (1.0%) in the R3R group, 54/5150 (1.0%) in the R3C group and 48/5153 (0.9%) in the C3C group (Fig. 1 and Supplementary Table 1). Of these, 96 received the fourth vaccine dose and 87 completed the study (Fig. 1).

In the RTS,S/AS01-vaccinated groups, HIV-infected participants were approximately equally distributed between the 6–12 weeks and 5–17 months age categories, while in the C3C group, two thirds of HIV-infected participants were in the older age category (Table 1). Supplementary Fig. 1 provides the age distribution at positive HIV test and does not indicate a major difference between groups. Apart from a trend for a higher proportion of girls in the R3C group, most baseline characteristics in the ITT population were similar between groups (Table 1). The highest number of HIV-infected participants were reported in Siaya, Kenya (56; 37%), Man-hiça, Mozambique (37; 24%), Kombewa, Kenya (19; 12%) and Lilongwe, Malawi (18; 12%) (Table 1). This corresponded to 3.5% of all participants enrolled in Siaya, 2.3% of those enrolled in Manhiça, 1.2% in Kombewa and 1.1% in Lilongwe (Supplementary Table 2). HIV testing rates varied substantially across centers and were highest in Siaya (55.5% of those enrolled at this center), Kombewa (19.6%), Lilongwe (16.8%) and Kilifi (15.6%) and lowest in Nanoro (0.08%) (Supplementary Table 2).

The per protocol population for immunogenicity comprised 56 RTS,S/AS01-vaccinated and 23 comparator-vaccinated participants identified as HIV-infected. Baseline characteristics of the per protocol population for immunogenicity were in line with those of the total ITT population of HIV-infected participants (Supplementary Table 3).

### 3.2 Safety

The median follow-up time between dose 1 and study end in the ITT population of HIV-infected participants was 39.3 months in the R3R group, 39.4 months in the R3C group and 38.3 months in the C3C group. During this period, SAEs were reported for 92.2% (95% CI: 81.1–97.8) of HIV-infected participants in the R3R group, 85.2% (72.9–93.4) in the R3C group and 87.5% (74.8–95.3) in the C3C group across the two age categories, with comparable rates in both age categories (Table 2). These percentages were similar when excluding SAEs with a Medical Dictionary for Regulatory Activities code referring to HIV infection (Table 2), indicating that most children experienced other SAEs in addition to HIV infection. The most commonly reported SAEs (aside from HIV infection) were pneumonia, gastroenteritis, different forms of malnutrition, malaria, and anemia, each reported at similar frequencies across groups (Supplementary Tables 4–6). Meningitis—which was identified as a safety signal after RTS,S/AS01 vaccination in the 5–17 months age category in the total population of the initial study [7,9,10,25]— was diagnosed for two (3.9%) HIV-infected participants in the R3R group, both in the 5–17 months age category, one (1.9%) in the R3C group in the 6–12 weeks age category, and one (2.1%) in the C3C group in the 5–17 months age category (Supplementary Tables 4–6). The etiology was identified for the case in the R3C group (pneumococcal meningitis).

Two SAEs were judged as related to vaccination. Both were cases of febrile convulsion in one participant in the R3R group in the 5–17 months age category; the first one occurred 2 days after administration of dose 3, the second one on the day of dose 4 administration.

**Fig. 2:**
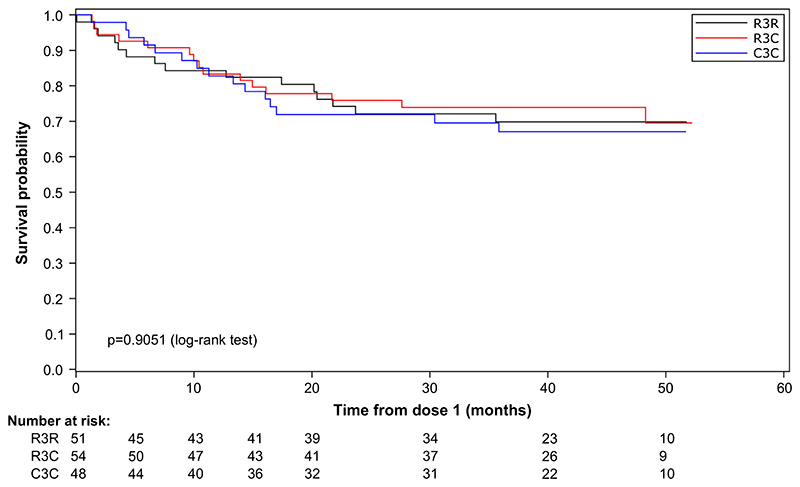
Survival curves (ITT population of HIV-infected participants). ITT, intent-to-treat; R3R, group receiving 4 doses of RTS,S/AS01; R3C, group receiving 3 doses of RTS,S/ AS01 plus 1 dose of comparator vaccine; C3C, group receiving 4 doses of comparator vaccine.

Fifteen HIV-infected participants in each group (R3R: 29.4%, R3C: 27.8%, C3C: 31.3%) died during the study (Table 2). No deaths were deemed related to vaccination. The most frequently reported fatal SAEs (aside from HIV infection) were lower respiratory tract infections and gastroenteritis (Supplementary Table 7). No statistically significant differences in survival duration were observed between RTS,S/AS01-vaccinated and comparator-vaccinated HIV-infected children (log-rank p values equaled 0.9051 for the overall population, 0.4025 for the 5–17 months and 0.7665 for the 6–12 weeks age category) (Fig. 2 and Supplementary Fig. 2).

In the overall population of the initial large phase III study, a *post hoc* analysis showed that all-cause mortality in RTS,S/AS01-vaccinated girls was ~1.8-fold higher than that in comparator-vaccinated girls, an imbalance not observed in boys [22,26]. A higher number (~1.9-fold) of deaths was also seen in RTS,S/AS01-vaccinated girls compared with comparator-vaccinated girls (and the opposite for boys) in the subset of HIV-infected children, but 95% CIs overlapped: 18/53 (34.0%; 95% CI: 21.5–48.3) RTS,S/ AS01-vaccinated girls (R3R + R3C) and 4/22 (18.2%; 5.2–40.3) comparator-vaccinated girls (C3C) died compared to 12/52 (23.1%; 12.5–36.8) RTS,S/AS01-vaccinated boys and 11/26 (42.3%; 23.4–63.1) comparator-vaccinated boys.

Three pIMDs were reported throughout the trial among HIV-infected children, none of which were considered related to vaccination: one participant in the R3R group and one in the C3C group each had encephalitis with a fatal outcome (onset 1 day post-dose 1 and 353 days post-dose 3, respectively) and one participant in the C3C group had Stevens-Johnson syndrome (onset 616 days post-dose 4) but the child recovered.

Thirty (28.6%) RTS,S/AS01-vaccinated (R3R + R3C) and 12 (25.0%) comparator-vaccinated HIV-infected participants experienced an SAE within 30 days after any of the first 3 vaccine doses. Most were HIV and opportunistic infections, with no apparent imbalances between groups (Table 2 and Supplementary Table 8). Four (12.1%) HIV-infected participants in the R3R group, three (8.6%) in the R3C group and one (3.6%) in the C3C group experienced an SAE within 30 days after the fourth dose (Table 2 and Supplementary Table 9).

### 3.3 Immunogenicity

One month post-dose 3, all HIV-infected RTS,S/AS01 recipients in the per protocol population for immunogenicity were seropositive for anti-CS antibodies in both age categories, compared to 23.5% (5–17 months) and 0.0% (6–12 weeks) in the C3C group (Table 3). While anti-CS antibody GMCs at screening were similarly low between groups (0.3 EU/mL), they were substantially higher in RTS,S/AS01-vaccinated than in comparator-vaccinated HIV-infected children 1 month post-dose 3 (188.7 vs 0.5 EU/mL; Table 3).

Results from the matched case-control analysis on the total vaccinated population of HIV-infected cases and immunogenicity controls indicated that anti-CS antibody GMCs after 3 doses of RTS,S/ AS01 were lower in vaccinated HIV-infected participants than in vaccinated immunogenicity controls who were HIV-negative or had an unknown HIV status (193.3 versus 491.5 EU/mL, p = 0.0001, Table 4).

**Fig. 3:**
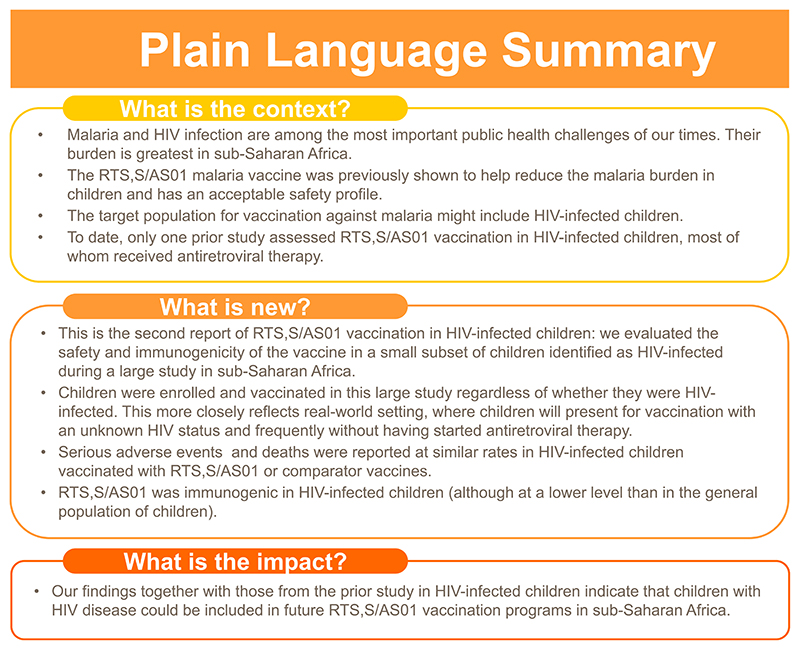
Plain language summary presenting the findings and highlighting their clinical relevance.

A “Plain Language Summary” section (Fig. 3) summarizes these findings and highlights their clinical relevance.

### 4 Discussion

Previous findings from our large phase III efficacy trial conducted across different malaria transmission settings in sub-Saharan Africa demonstrated that the RTS,S/AS01 vaccine can reduce clinical and severe malaria rates when administered as a 3- or 4-dose regimen in children [7–10]. The rates of SAEs reported in the overall population in this trial were similar in the RTS,S/ AS01-vaccinated and MenC-CRM- or rabies-vaccinated comparator groups [7–10,22]. In the current analyses, we have shown that for the subset of HIV-infected children in this trial (approximately 1% of the overall study population), the incidence of SAEs was also similar between vaccine groups, both when assessed over the entire study period and within 30 days after vaccination. No significant differences in mortality were observed between groups although samples sizes were small. RTS,S/AS01 was immunogenic in HIV-infected participants, but antibody concentrations were lower than in matched immunogenicity controls with an unknown or negative HIV status.

A prior study designed to evaluate the RTS,S/AS01 vaccine in HIV-infected children also showed similar rates of SAEs in RTS,S/ AS01 and rabies comparator vaccine recipients [13]. This agrees with other non-replicating vaccines that are not generally associated with an increased risk of complications when administered to HIV-infected children, in contrast to some live-attenuated vaccines [27–30].

Our results allow a direct comparison of the safety outcomes in HIV-infected children with those in the overall study population (HIV-infected and uninfected) in the same settings. While no imbalance in the SAE incidence was found between vaccine groups in either of these populations, the incidence of SAEs in the HIV-infected subset was substantially higher than that in the overall study population, as expected (83–94% versus 24–28% across study and age groups throughout the study) [10]. This difference was mostly due to the higher incidence of common childhood infectious diseases and other conditions for which HIV infection increases susceptibility (such as pneumonia, gastroenteritis, malaria and different forms of malnutrition) [3,31–34].

The incidence of SAEs and fatal SAEs in our HIV-infected subset of children was higher than that observed in the prior study evaluating RTS,S/AS01 vaccination in HIV-infected children, in which 41% of participants had at least one SAE and 5% died [13]. Participants in that study were diagnosed as HIV-infected before enrollment and most received ART (to delay disease progression) and daily cotrimoxazole (to prevent opportunistic infections) before or soon after enrollment. By contrast, as no systematic HIV screening was done in our large phase III study and testing to confirm HIV infection was performed based on clinical suspicion, HIV treatment likely started at a more advanced stage of HIV in participants in our study. In addition, the prior study had a follow-up time of 14 months after first vaccination compared to a median follow-up time of approximately 39 months in our current analyses.

Two SAEs (both febrile convulsions reported for the same child in the 5–17 months age category) were considered related to vaccination. This is in line with safety data for the overall study population which showed an increased risk of febrile convulsion, particularly during the first 2–3 days after RTS,S/AS01 vaccination in the 5–17 months age category [22]. Febrile convulsion was also the only vaccination-related SAE reported in the prior RTS,S/AS01 study in HIV-infected children [13].

In addition to an increased risk of febrile convulsion, a higher number of meningitis cases reported as SAE was observed in RTS, S/AS01 compared to comparator vaccinees in the 5–17 months age category in the overall study population of our initial large phase III trial [7,9,10]. No such imbalance seemed apparent in the HIV-infected subset. However, the number of meningitis cases in this subset was low.

In the prior study in HIV-infected children, more SAEs were observed within 30 days after RTS,S/AS01 than after rabies vaccination, which the authors partially attributed to a higher number of pneumonia cases in the RTS,S/AS01 group. However, they noted no clustering in time-to-onset and the imbalance did not remain until study end. In the HIV-infected subset in our current analyses, there was no apparent imbalance in the incidence of pneumonia within 30 days after vaccination or over the entire follow-up period.

The numerical imbalance in overall mortality between RTS,S/ AS01 and comparator-vaccinated girls in the HIV-infected population assessed here contributed to, but does not fully explain the imbalance seen in the overall population of our initial large phase III study [22].

Three doses of RTS,S/AS01 were immunogenic in our subset of HIV-infected children, but anti-CS antibody GMCs were lower than in the matched immunogenicity control cohort (including children with an unknown or negative HIV status). Likewise, the prior RTS,S/ AS01 study in HIV-infected children reported lower anti-CS antibody GMCs 1 month post-dose 3 in HIV-infected participants compared to children enrolled at the same study centers in our large phase III efficacy study [13]. The impact of this lower immune response on the vaccine’s protective efficacy is unknown. However, in the prior study in HIV-infected children, RTS,S/AS01 vaccinees experienced 37% fewer episodes of clinical malaria as well as fewer severe malaria episodes and malaria-related hospitalizations than rabies vaccinees. While these differences were not statistically significant (the study was not powered to measure vaccine efficacy against these outcomes), these results indicated that, despite the lower immunogenicity, RTS,S/AS01 might still provide some protection against malaria in HIV-infected children on ART [13]. A reduced immunological response to vaccination in HIV-infected children (particularly when not on ART) has been observed for several other childhood vaccines and is likely a result of HIV infection impairing both CD4+ T cell and memory B cell responses [28,35–37].

Our analysis on the HIV-infected subset of the large phase III RTS,S/AS01 trial has some limitations. Firstly, HIV testing was not a trial procedure and was thus not done systematically on all participants. As a result, some HIV-infected children might not have been identified and—because HIV-testing practices and testing rates varied among centers—different proportions may have been missed in the different centers. The real impact of HIV infection on the anti-CS immune response may have been overestimated if mostly more advanced stages of HIV were clinically diagnosed and included in the HIV-infected subset. Alternatively, the impact may have been underestimated if unknown HIV-infected children were included as immunogenicity controls. Secondly, HIV-infected individuals were not randomized to the RTS,S/AS01 or comparator vaccine groups. However, approximately equal proportions of HIV-infected children were identified in all groups and most baseline characteristics were comparable between the groups in the HIV-infected subset. Thirdly, the lack of information on ART use and HIV disease progression (CD4 counts and viral load), both of which may influence safety and immunogenicity results, are limitations in fully understanding the generalizability of the study findings. This was however assessed in detail in the prior study in HIV-infected children [13].

Our results contribute to the overall risk-benefit assessment of the RTS,S/AS01 vaccine. In contrast to the prior study, which only enrolled HIV-infected children [13], our results allow a direct comparison of the safety and immunogenicity of RTS,S/AS01 in HIV-infected children with those in the overall study population (HIV-infected and uninfected) in the same settings. The prior study in HIV-infected children evaluated a 3-dose vaccination course (0, 1, 2 months) with a follow-up of 14 months [13] while the current analyses also included the safety of a fourth dose given 18 months after dose 3 and had a follow-up of more than 3 years after first vaccination. The prior study was conducted at two sites in Kenya, while our current analyses also provided data on children in other sub-Saharan countries. HIV testing was not a study procedure in our large phase III trial and thus was done according to daily practice in the different study centers. This might therefore more closely reflect a real-life situation where children may be vaccinated without knowledge of their HIV status and/or without having started ART.

## 5 Conclusions

Our analyses on the subset of children identified as HIV-infected during the large phase III RTS,S/AS01 trial in sub-Saharan Africa indicate that the incidence of SAEs and deaths in RTS,S/AS01-vaccinated HIV-infected children is similar to that in MenC-CRM- or rabies-vaccinated HIV-infected children. RTS,S/AS01 is immunogenic in this population, although at a lower level than in the general population of children. This, together with results from the prior RTS,S/AS01 study in HIV-infected children, suggests that RTS,S/AS01 would be expected to provide some protection against malaria in HIV-infected children. Based on these data, we see no reason why the RTS,S/AS01 vaccine would be contraindicated in infants and children with stage I/II HIV disease, which is in line with recommendations for other non-replicating, recombinant vaccines [38].

## Supplementary Material

Supplementary Material

## Figures and Tables

**Table 1:** Baseline characteristics of the ITT population of HIV-infected participants.

Characteristic	R3R group N = 51	R3C group N = 54	C3C group N = 48
**Age category**			
5–17 months, n (%)	24 (47.1)	29 (53.7)	32 (66.7)
6–12 weeks, n (%)	27 (52.9)	25 (46.3)	16 (33.3)
**Mean age at dose 1±SD**			
5–17 months, months	10.3 ± 3.4	9.7 ± 4.0	10.8 ± 3.8
6–12 weeks, weeks	7.6 ± 1.5	7.6 ± 1.6	7.5 ± 1.5
**Mean age at HIV positive test ± SD**			
5–17 months, months	17.9 ± 7.5	22.4 ± 13.1	17.7 ± 12.3
6–12 weeks, months	9.1 ± 7.8	10.0 ± 8.8	15.6 ± 9.9
Both age categories, months	13.8 ± 8.8	17.1 ± 12.8	17.5 ± 11.6
**Female sex, n (%)**	19 (37.3)	34 (63.0)	22 (45.8)
**Mean height-for-age z-score ± SD**	—1.6 ± 1.1	—1.7 ± 1.5^a^	—1.6 ± 1.3
**Mean weight-for-age z-score ± SD**	—1.1 ± 1.2^b^	—1.4 ± 1.4	—1.1 ± 1.1
**Mean Hb ± SD, g/dL**	10.2 ± 1.7	10.3 ± 1.6	10.2 ± 1.7
**Moderate anemia** ^c^ **, n (%)**	4 (7.8)	3 (5.6)	2 (4.2)
**Study center, n (%)**			
Agogo, Ghana	2 (3.9)	0 (0.0)	1 (2.1)
Bagamoyo, Tanzania	2 (3.9)	1 (1.9)	1 (2.1)
Kilifi, Kenya	0 (0.0)	2 (3.7)	2 (4.2)
Kintampo, Ghana	0 (0.0)	5 (9.3)	3 (6.3)
Kombewa, Kenya	8 (15.7)	5 (9.3)	6 (12.5)
Korogwe, Tanzania	1 (2.0)	2 (3.7)	0 (0.0)
Lambaréné, Gabon	0 (0.0)	0 (0.0)	1 (2.1)
Lilongwe, Malawi	3 (5.9)	9 (16.7)	6 (12.5)
Manhiça, Mozambique	11 (21.6)	12 (22.2)	14(29.2)
Nanoro, Burkina Faso	0 (0.0)	0 (0.0)	0 (0.0)
Siaya, Kenya	24 (47.1)	18 (33.3)	14(29.2)


ITT, intent-to-treat; R3R, group receiving 4 doses of RTS,S/AS01; R3C, group receiving 3 doses of RTS,S/AS01 plus 1 dose of comparator vaccine; C3C, group receiving 4 doses of comparator vaccine; N, number of participants in the ITT population of HIV-infected participants; n (%), number (percentage) of participants in a given category; SD, standard deviation; Hb, hemoglobin.

a Height-for-age z-score available for 52 participants in the R3C group.

b Weight-for-age z-score available for 50 participants in the R3R group.

c Hb < 8g/dL.

**Table 2:** Number and percentage of HIV-infected participants for whom serious adverse events were reported during the specified periods, overall and by age category (ITT population of HIV-infected participants).

	R3R		R3C		C3C	
	N	n (%, 95% CI)	N	n (%, 95% CI)	N	n (%, 95% CI)
**At least one SAE between dose 1 and study end**						
Total	51	47 (92.2, 81.1-97.8)	54	46 (85.2, 72.9-93.4)	48	42 (87.5, 74.8-95.3)
5–17 months	24	22 (91.7, 73.0–99.0)	29	24 (82.8, 64.2–94.2)	32	27 (84.4, 67.2–94.7)
6–12 weeks	27	25 (92.6, 75.7–99.1)	25	22 (88.0, 68.8–97.5)	16	15 (93.8, 69.8–99.8)
**At least one SAE (excluding HIV infection**^a^) **between dose 1 and study end**						
Total	51	43 (84.3, 71.4–93.0)	54	44 (81.5, 68.6–90.7)	48	40 (83.3, 69.8–92.5)
5–17 months	24	21 (87.5, 67.6–97.3	29	23 (79.3, 60.3–92.0)	32	27 (84.4, 67.2–94.7)
6–12 weeks	27	22 (81.5, 61.9–93.7)	25	21 (84.0, 63.9–95.5)	16	13 (81.3, 54.4–96.0)
**Fatalities between dose 1 and study end**						
Total	51	15 (29.4, 17.5–43.8)	54	15 (27.8, 16.5–41.6)	48	15 (31.3, 18.7–46.3)
5–17 months	24	6 (25.0, 9.8–46.7)	29	6 (20.7, 8.0–39.7)	32	11 (34.4, 18.6–53.2)
6–12 weeks	27	9 (33.3, 16.5–54.0)	25	9 (36.0, 18.0–57.5)	16	4 (25.0, 7.3–52.4)
**At least one SAE within 30 days after any of the first 3 doses**						
Total	51	14 (27.5, 15.9–41.7)	54	16 (29.6, 18.0–43.6)	48	12 (25.0, 13.6–39.6)
5–17 months	24	7 (29.2, 12.6–51.1)	29	6 (20.7, 8.0–39.7)	32	10(31.3, 16.1–50.0)
6–12 weeks	27	7 (25.9, 11.1–46.3)	25	10 (40.0, 21.1–61.3)	16	2 (12.5, 1.6–38.3)
**At least one SAE within 30 days after dose 4**						
Total	33	4 (12.1, 3.4–28.2)	35	3 (8.6, 1.8–23.1)	28	1 (3.6, 0.1–18.3)
5–17 months	17	2 (11.8, 1.5–36.4)	22	2 (9.1, 1.1–29.2)	20	1 (5.0,0.1–24.9)
6–12 weeks	16	2 (12.5, 1.6–38.3)	13	1 (7.7,0.2–36.0)	8	0 (0.0, 0.0–36.9)

ITT, intent-to-treat; R3R, group receiving 4 doses of RTS,S/AS01; R3C, group receiving 3 doses of RTS,S/AS01 plus 1 dose of comparator vaccine; C3C, group receiving 4 doses of comparator vaccine; N, number of participants with at least one administered vaccine dose; for SAEs reported within 30 days after dose 4, N represents the number of participants who received dose 4; n (%), number (percentage) of participants reporting the event at least once; CI, confidence interval; SAE, serious adverse event.

a Excluding SAEs with Medical Dictionary for Regulatory Activities codes referring to HIV infection: “HIV infection”, “HIV infection WHO clinical stage II”, “HIV infection WHO clinical stage III” and “HIV infection WHO clinical stage IV”.

**Table 3:** Seropositivity rates and geometric mean concentrations for anti-CS antibodies at screening and 1 month post-dose 3 in RTS,S/AS01- and comparator-vaccinated HIV-infected participants (per protocol population for immunogenicity of HIV-infected participants).

Time point	R3C + R3R			C3C		
	N	% with anti-CS ≥ 0.5 EU/mL (95% CI)	GMC (95% CI), EU/mL	N	% with anti-CS ≥ 0.5 EU/mL (95% CI)	GMC (95% CI), EU/mL
**Pooled across age categories**					
Screening	53	15.1 (6.7–27.6)	0.3 (0.3–0.4)	21	23.8 (8.2–47.2)	0.3 (0.3–0.4)
1 month post-dose 3	53	100 (93.3–100)	188.7 (115.2–309.0)	22	18.2 (5.2–40.3)	0.5 (0.2–1.1)
**5–17 months**						
Screening	28	14.3 (4.0–32.7)	0.3 (0.2–0.5)	16	31.3 (11.0–58.7)	0.4 (0.3–0.5)
1 month post-dose 3	29	100 (88.1–100)	264.7 (137.5–509.6)	17	23.5 (6.8–49.9)	0.5 (0.2–1.7)
**6–12 weeks**						
Screening	25	16.0 (4.5–36.1)	0.3 (0.2–0.4)	5	0.0 (0.0–52.2)	0.3 (0.3–0.3)
1 month post-dose 3	24	100 (85.8–100)	125.3 (58.1–270.3)	5	0.0 (0.0–52.2)	0.3 (0.3–0.3)

CS, circumsporozoite; R3R, group receiving 4 doses of RTS,S/AS01; R3C, group receiving 3 doses of RTS,S/AS01 plus 1 dose of comparator vaccine; C3C, group receiving 4 doses of comparator vaccine; N, number of participants in the per protocol population with available results, including only those confirmed as HIV-infected by 2 May 2012 (data lock point when most children had performed their study month 20 visit); % with anti-CS ≥ 0.5 EU/mL, percentage of participants with an anti-CS antibody concentration ≥ 0.5 EU/mL; EU, enzyme-linked immunosorbent assay unit; CI, confidence interval; GMC, geometric mean concentration.

**Table 4:** Seropositivity rates and geometric mean concentrations for anti-CS antibodies at screening and 1 month post-dose 3 in RTS,S/AS01-vaccinated participants in the matched casecontrol analysis (total vaccinated population of HIV-infected cases and their matched RTS,S/AS01-vaccinated immunogenicity controls).

Time point	R3R + R3C, HIV-infected			R3R + R3C, immunogenicity control^a^		
	N	% with anti-CS ≥ 0.5 EU/mL (95% CI)	GMC (95% CI), EU/mL	N	% with anti-CS ≥ 0.5 EU/mL (95% CI)	GMC (95% CI), EU/mL
Screening	60	16.7 (8.3–28.5)	0.3 (0.3–0.4)	58	19.0 (9.9–31.4)	0.3 (0.3–0.4)
1 month post-dose 3	61	100 (94.1–100)	193.3 (124.1–301.0)	61	100 (94.1–100)	491.5 (406.3–594.6)

p values for the comparison of log-transformed means of anti-CS antibodies were 0.8316 at screening and 0.0001 at 1 month post-dose 3.

CS, circumsporozoite; R3R, group receiving 4 doses of RTS,S/AS01; R3C, group receiving 3 doses of RTS,S/AS01 plus 1 dose of comparator vaccine; N, number of participants in the total vaccinated population with available results, including only those confirmed as HIV-infected or not identified as HIV-infected by 2 May 2012 (data lock point when most children had performed their study month 20 visit); % with anti-CS ≥ 0.5 EU/mL, percentage of participants with an anti-CS antibody concentration ≥ 0.5 EU/mL; EU, enzyme-linked immunosorbent assay unit; CI, confidence interval; GMC, geometric mean concentration.

a Immunogenicity controls were RTS,S/AS01-vaccinated with either an unknown or negative HIV status.

## Abbreviations

ART: antiretroviral therapy
AS: adjuvant system
CS: circumsporozoite
CI: confidence interval
ELISA: enzyme-linked immunosorbent assay
EU: ELISA unit
GMC: geometric mean concentration
HBsAg: hepatitis B surface antigen
HIV: human immunodeficiency virus
ITT: intent-to-treat
LAR: legally authorized representative
MenC-CRM: meningococcal C CRM197 conjugate vaccine
PCR: polymerase chain reaction
pIMD: potential immune-mediated disorder
SAE: serious adverse event
WHO: World Health Organization
