# Supplementary material for: Safety and immunogenicity of the RTS,S/AS01 malaria vaccine in infants and children identified as HIV-infected during a randomized trial in sub-Saharan Africa
Source: Vaccine. Author manuscript; Available in PMC 2022 Sep 7. (PMC7613311; doi:10.1016/j.vaccine.2019.10.077)
Supplement: Supplementary Material [file EMS152520-supplement-Supplementary_Material.docx]

**Supplementary material**

**Supplementary table 1. Number and percentage of participants reported to have been tested for HIV at least once from enrollment to study end, by study group and age category (ITT population)**

| **Age category** | **R3R**  **N=621** | **R3C**  **N=677** | **C3C**  **N=655** |
| --- | --- | --- | --- |
| 5–17 months, n (%) | 352 (56.7) | 403 (59.5) | 379 (57.9) |
| 6–12 weeks, n (%) | 269 (43.3) | 274 (40.5) | 276 (42.1) |

ITT, intent-to-treat; R3R, group receiving 4 doses of RTS,S/AS01; R3C, group receiving 3 doses of RTS,S/AS01 plus 1 dose of comparator vaccine; C3C, group receiving 4 doses of comparator vaccine; N, number of participants tested for HIV in each study group; n (%), number (percentage) of those tested in each age category.

**Supplementary table 2. Number and percentage of participants reported to have been tested for HIV and confirmed as HIV-infected from enrollment to study end, by study center (ITT population)**

| **Study center** | **Total enrolled,**  **N** | **Tested,**  **n (%)** | **HIV-infected,**  **n (%)** |
| --- | --- | --- | --- |
| **Agogo, Ghana** | 1288 | 18 (1.40) | 3 (0.23) |
| **Bagamoyo, Tanzania** | 1705 | 94 (5.51) | 4 (0.23) |
| **Kilifi, Kenya** | 904 | 141 (15.60) | 4 (0.44) |
| **Kintampo, Ghana** | 1333 | 18 (1.35) | 8 (0.60) |
| **Kombewa, Kenya** | 1631 | 319 (19.56) | 19 (1.16) |
| **Korogwe, Tanzania** | 1505 | 7 (0.47) | 3 (0.20) |
| **Lambaréné, Gabon** | 930 | 44 (4.73) | 1 (0.11) |
| **Lilongwe, Malawi** | 1626 | 273 (16.79) | 18 (1.11) |
| **Manhiça, Mozambique** | 1637 | 139 (8.49) | 37 (2.26) |
| **Nanoro, Burkina Faso** | 1281 | 1 (0.08) | 0 (0.00) |
| **Siaya, Kenya** | 1619 | 899 (55.53) | 56 (3.46) |
| **Total** | 15459 | 1953 (12.63) | 153 (0.99) |

ITT, intent-to-treat; N, total number of participants enrolled per center; n (%), number (percentage) of participants tested for or confirmed as HIV-infected per center.

**Supplementary table 3. Baseline characteristics of the per protocol population for immunogenicity of HIV-infected participants**

| **Characteristic** | **R3R + R3C group**  **N=56** | **C3C group**  **N=23** |
| --- | --- | --- |
| **Age category**  5–17 months, n (%)  6–12 weeks, n (%) | 29 (51.8)  27 (48.2) | 18 (78.3)  5 (21.7) |
| **Mean age at dose 1 ± SD**  5–17 months, months  6–12 weeks, weeks | 10.6 ± 3.6  7.3 ± 1.4 | 10.2 ± 3.9  7.2 ± 1.3 |
| **Female sex, n (%)** | 23 (41.1) | 9 (39.1) |
| **Mean height-for-age z-score ± SD** | -1.8 ± 1.1 | -1.7 ± 1.2 |
| **Mean weight-for-age z-score ± SD** | -1.1 ± 1.2 | -1.2 ± 1.2 |
| **Mean Hb ± SD, g/dL** | 10.4 ± 1.7 | 9.8 ± 1.5 |
| **Moderate anemia^a^, n (%)** | 4 (7.1) | 2 (8.7) |

R3R, group receiving 4 doses of RTS,S/AS01; R3C, group receiving 3 doses of RTS,S/AS01 plus 1 dose of comparator vaccine; C3C, group receiving 4 doses of comparator vaccine; N, number of participants in the per protocol population for immunogenicity of HIV-infected participants including only those confirmed as HIV-infected by 2 May 2012 (data lock point when most children had performed their study month 20 visit); n (%), number (percentage) of participants in a given category; SD, standard deviation; Hb, hemoglobin.

^a^Hb <8 g/dL.

**Supplementary table 4. Number and percentage of HIV-infected participants for whom serious adverse events were reported between dose 1 and study end, listed by Medical Dictionary for Regulatory Activities system organ class and preferred term (ITT population of HIV-infected participants)**

|  | | **R3R  N=51** | | | |  | **R3C  N=54** | | | |  | **C3C  N=48** | | | |
| --- | --- | --- | --- | --- | --- | --- | --- | --- | --- | --- | --- | --- | --- | --- | --- |
|  | |  | | **95% CI** | |  |  | | **95% CI** | |  |  | | **95% CI** | |
| **Primary System Organ Class (CODE)** | **Preferred Term (CODE)** | **n** | **%** | **LL** | **UL** |  | **n** | **%** | **LL** | **UL** |  | **n** | **%** | **LL** | **UL** |
| At least one SAE |  | 47 | 92.2 | 81.1 | 97.8 |  | 46 | 85.2 | 72.9 | 93.4 |  | 42 | 87.5 | 74.8 | 95.3 |
| Blood and lymphatic system disorders (10005329) | Anemia (10002034) | 9 | 17.6 | 8.4 | 30.9 |  | 11 | 20.4 | 10.6 | 33.5 |  | 14 | 29.2 | 17.0 | 44.1 |
|  | Disseminated intravascular coagulation (10013442) | 1 | 2.0 | 0.0 | 10.4 |  | 0 | 0.0 | 0.0 | 6.6 |  | 0 | 0.0 | 0.0 | 7.4 |
|  | Neutropenia (10029354) | 0 | 0.0 | 0.0 | 7.0 |  | 1 | 1.9 | 0.0 | 9.9 |  | 0 | 0.0 | 0.0 | 7.4 |
| Cardiac disorders (10007541) | Pericardial effusion (10034474) | 0 | 0.0 | 0.0 | 7.0 |  | 0 | 0.0 | 0.0 | 6.6 |  | 1 | 2.1 | 0.1 | 11.1 |
| Congenital, familial and genetic disorders (10010331) | Sickle cell anemia with crisis (10040642) | 0 | 0.0 | 0.0 | 7.0 |  | 1 | 1.9 | 0.0 | 9.9 |  | 0 | 0.0 | 0.0 | 7.4 |
| Gastrointestinal disorders (10017947) | Colitis (10009887) | 0 | 0.0 | 0.0 | 7.0 |  | 0 | 0.0 | 0.0 | 6.6 |  | 1 | 2.1 | 0.1 | 11.1 |
|  | Enteritis (10014866) | 0 | 0.0 | 0.0 | 7.0 |  | 1 | 1.9 | 0.0 | 9.9 |  | 2 | 4.2 | 0.5 | 14.3 |
|  | Vomiting (10047700) | 1 | 2.0 | 0.0 | 10.4 |  | 0 | 0.0 | 0.0 | 6.6 |  | 0 | 0.0 | 0.0 | 7.4 |
| General disorders and administration site conditions (10018065) | Hypothermia (10021113) | 1 | 2.0 | 0.0 | 10.4 |  | 0 | 0.0 | 0.0 | 6.6 |  | 0 | 0.0 | 0.0 | 7.4 |
|  | Pyrexia (10037660) | 1 | 2.0 | 0.0 | 10.4 |  | 1 | 1.9 | 0.0 | 9.9 |  | 1 | 2.1 | 0.1 | 11.1 |
| Hepatobiliary disorders (10019805) | Hepatitis acute (10019727) | 0 | 0.0 | 0.0 | 7.0 |  | 1 | 1.9 | 0.0 | 9.9 |  | 0 | 0.0 | 0.0 | 7.4 |
| Immune system disorders (10021428) | Anaphylactic reaction (10002198) | 0 | 0.0 | 0.0 | 7.0 |  | 0 | 0.0 | 0.0 | 6.6 |  | 1 | 2.1 | 0.1 | 11.1 |
|  | Immune reconstitution inflammatory syndrome (10065042) | 0 | 0.0 | 0.0 | 7.0 |  | 0 | 0.0 | 0.0 | 6.6 |  | 1 | 2.1 | 0.1 | 11.1 |
| Infections and infestations (10021881) | Abscess (10000269) | 0 | 0.0 | 0.0 | 7.0 |  | 1 | 1.9 | 0.0 | 9.9 |  | 0 | 0.0 | 0.0 | 7.4 |
|  | Abscess limb (10050473) | 0 | 0.0 | 0.0 | 7.0 |  | 0 | 0.0 | 0.0 | 6.6 |  | 1 | 2.1 | 0.1 | 11.1 |
|  | Aids dementia complex (10065040) | 0 | 0.0 | 0.0 | 7.0 |  | 0 | 0.0 | 0.0 | 6.6 |  | 1 | 2.1 | 0.1 | 11.1 |
|  | Arthritis bacterial (10053555) | 0 | 0.0 | 0.0 | 7.0 |  | 1 | 1.9 | 0.0 | 9.9 |  | 0 | 0.0 | 0.0 | 7.4 |
|  | Bronchiolitis (10006448) | 1 | 2.0 | 0.0 | 10.4 |  | 0 | 0.0 | 0.0 | 6.6 |  | 0 | 0.0 | 0.0 | 7.4 |
|  | Bronchitis (10006451) | 0 | 0.0 | 0.0 | 7.0 |  | 0 | 0.0 | 0.0 | 6.6 |  | 1 | 2.1 | 0.1 | 11.1 |
|  | Bronchopneumonia (10006469) | 3 | 5.9 | 1.2 | 16.2 |  | 7 | 13.0 | 5.4 | 24.9 |  | 4 | 8.3 | 2.3 | 20.0 |
|  | Cellulitis of male external genital organ (10064929) | 1 | 2.0 | 0.0 | 10.4 |  | 0 | 0.0 | 0.0 | 6.6 |  | 0 | 0.0 | 0.0 | 7.4 |
|  | Conjunctivitis (10010741) | 1 | 2.0 | 0.0 | 10.4 |  | 1 | 1.9 | 0.0 | 9.9 |  | 0 | 0.0 | 0.0 | 7.4 |
|  | Disseminated tuberculosis (10013453) | 1 | 2.0 | 0.0 | 10.4 |  | 0 | 0.0 | 0.0 | 6.6 |  | 1 | 2.1 | 0.1 | 11.1 |
|  | Dysentery (10051402) | 0 | 0.0 | 0.0 | 7.0 |  | 2 | 3.7 | 0.5 | 12.7 |  | 0 | 0.0 | 0.0 | 7.4 |
|  | Encephalitis (10014581) | 1 | 2.0 | 0.0 | 10.4 |  | 0 | 0.0 | 0.0 | 6.6 |  | 1 | 2.1 | 0.1 | 11.1 |
|  | Enterococcal sepsis (10054221) | 1 | 2.0 | 0.0 | 10.4 |  | 0 | 0.0 | 0.0 | 6.6 |  | 0 | 0.0 | 0.0 | 7.4 |
|  | Escherichia sepsis (10015296) | 1 | 2.0 | 0.0 | 10.4 |  | 0 | 0.0 | 0.0 | 6.6 |  | 0 | 0.0 | 0.0 | 7.4 |
|  | Gastroenteritis (10017888) | 22 | 43.1 | 29.3 | 57.8 |  | 23 | 42.6 | 29.2 | 56.8 |  | 17 | 35.4 | 22.2 | 50.5 |
|  | Gastroenteritis salmonella (10017914) | 1 | 2.0 | 0.0 | 10.4 |  | 1 | 1.9 | 0.0 | 9.9 |  | 0 | 0.0 | 0.0 | 7.4 |
|  | Gastroenteritis shigella (10017915) | 0 | 0.0 | 0.0 | 7.0 |  | 0 | 0.0 | 0.0 | 6.6 |  | 1 | 2.1 | 0.1 | 11.1 |
|  | Gastrointestinal candidiasis (10017938) | 0 | 0.0 | 0.0 | 7.0 |  | 1 | 1.9 | 0.0 | 9.9 |  | 0 | 0.0 | 0.0 | 7.4 |
|  | Groin abscess (10050269) | 1 | 2.0 | 0.0 | 10.4 |  | 0 | 0.0 | 0.0 | 6.6 |  | 0 | 0.0 | 0.0 | 7.4 |
|  | Helminthic infection (10061201) | 0 | 0.0 | 0.0 | 7.0 |  | 1 | 1.9 | 0.0 | 9.9 |  | 2 | 4.2 | 0.5 | 14.3 |
|  | HIV associated nephropathy (10070737) | 0 | 0.0 | 0.0 | 7.0 |  | 0 | 0.0 | 0.0 | 6.6 |  | 1 | 2.1 | 0.1 | 11.1 |
|  | HIV infection (10020161)^a^ | 38 | 74.5 | 60.4 | 85.7 |  | 34 | 63.0 | 48.7 | 75.7 |  | 26 | 54.2 | 39.2 | 68.6 |
|  | HIV infection who clinical stage ii (10064447)^a^ | 0 | 0.0 | 0.0 | 7.0 |  | 0 | 0.0 | 0.0 | 6.6 |  | 1 | 2.1 | 0.1 | 11.1 |
|  | HIV infection who clinical stage iii (10064448)^a^ | 1 | 2.0 | 0.0 | 10.4 |  | 1 | 1.9 | 0.0 | 9.9 |  | 0 | 0.0 | 0.0 | 7.4 |
|  | HIV infection who clinical stage iv (10064456)^a^ | 1 | 2.0 | 0.0 | 10.4 |  | 1 | 1.9 | 0.0 | 9.9 |  | 0 | 0.0 | 0.0 | 7.4 |
|  | Impetigo (10021531) | 0 | 0.0 | 0.0 | 7.0 |  | 0 | 0.0 | 0.0 | 6.6 |  | 2 | 4.2 | 0.5 | 14.3 |
|  | Infected skin ulcer (10021784) | 0 | 0.0 | 0.0 | 7.0 |  | 1 | 1.9 | 0.0 | 9.9 |  | 0 | 0.0 | 0.0 | 7.4 |
|  | Klebsiella sepsis (10054160) | 1 | 2.0 | 0.0 | 10.4 |  | 0 | 0.0 | 0.0 | 6.6 |  | 0 | 0.0 | 0.0 | 7.4 |
|  | Lobar pneumonia (10024738) | 8 | 15.7 | 7.0 | 28.6 |  | 4 | 7.4 | 2.1 | 17.9 |  | 7 | 14.6 | 6.1 | 27.8 |
|  | Lymph node abscess (10050167) | 0 | 0.0 | 0.0 | 7.0 |  | 1 | 1.9 | 0.0 | 9.9 |  | 0 | 0.0 | 0.0 | 7.4 |
|  | Lymph node tuberculosis (10025183) | 0 | 0.0 | 0.0 | 7.0 |  | 0 | 0.0 | 0.0 | 6.6 |  | 1 | 2.1 | 0.1 | 11.1 |
|  | Malaria (10025487) | 12 | 23.5 | 12.8 | 37.5 |  | 18 | 33.3 | 21.1 | 47.5 |  | 11 | 22.9 | 12.0 | 37.3 |
|  | Meningitis (10027199) | 2 | 3.9 | 0.5 | 13.5 |  | 0 | 0.0 | 0.0 | 6.6 |  | 1 | 2.1 | 0.1 | 11.1 |
|  | Meningitis pneumococcal (10027253) | 0 | 0.0 | 0.0 | 7.0 |  | 1 | 1.9 | 0.0 | 9.9 |  | 0 | 0.0 | 0.0 | 7.4 |
|  | Oral candidiasis (10030963) | 3 | 5.9 | 1.2 | 16.2 |  | 3 | 5.6 | 1.2 | 15.4 |  | 2 | 4.2 | 0.5 | 14.3 |
|  | Oropharyngeal candidiasis (10050346) | 1 | 2.0 | 0.0 | 10.4 |  | 1 | 1.9 | 0.0 | 9.9 |  | 0 | 0.0 | 0.0 | 7.4 |
|  | Osteomyelitis (10031252) | 0 | 0.0 | 0.0 | 7.0 |  | 0 | 0.0 | 0.0 | 6.6 |  | 1 | 2.1 | 0.1 | 11.1 |
|  | Otitis media (10033078) | 2 | 3.9 | 0.5 | 13.5 |  | 5 | 9.3 | 3.1 | 20.3 |  | 5 | 10.4 | 3.5 | 22.7 |
|  | Parotitis (10034038) | 0 | 0.0 | 0.0 | 7.0 |  | 2 | 3.7 | 0.5 | 12.7 |  | 0 | 0.0 | 0.0 | 7.4 |
|  | Periorbital cellulitis (10057182) | 0 | 0.0 | 0.0 | 7.0 |  | 0 | 0.0 | 0.0 | 6.6 |  | 1 | 2.1 | 0.1 | 11.1 |
|  | Pharyngitis (10034835) | 1 | 2.0 | 0.0 | 10.4 |  | 0 | 0.0 | 0.0 | 6.6 |  | 0 | 0.0 | 0.0 | 7.4 |
|  | Pneumococcal sepsis (10054047) | 2 | 3.9 | 0.5 | 13.5 |  | 3 | 5.6 | 1.2 | 15.4 |  | 0 | 0.0 | 0.0 | 7.4 |
|  | Pneumocystis jirovecii pneumonia (10073755) | 5 | 9.8 | 3.3 | 21.4 |  | 1 | 1.9 | 0.0 | 9.9 |  | 1 | 2.1 | 0.1 | 11.1 |
|  | Pneumonia (10035664) | 18 | 35.3 | 22.4 | 49.9 |  | 25 | 46.3 | 32.6 | 60.4 |  | 21 | 43.8 | 29.5 | 58.8 |
|  | Pneumonia pneumococcal (10035728) | 0 | 0.0 | 0.0 | 7.0 |  | 1 | 1.9 | 0.0 | 9.9 |  | 0 | 0.0 | 0.0 | 7.4 |
|  | Pneumonia viral (10035737) | 0 | 0.0 | 0.0 | 7.0 |  | 1 | 1.9 | 0.0 | 9.9 |  | 0 | 0.0 | 0.0 | 7.4 |
|  | Pulmonary tuberculosis (10037440) | 8 | 15.7 | 7.0 | 28.6 |  | 4 | 7.4 | 2.1 | 17.9 |  | 0 | 0.0 | 0.0 | 7.4 |
|  | Pyoderma (10037632) | 0 | 0.0 | 0.0 | 7.0 |  | 0 | 0.0 | 0.0 | 6.6 |  | 1 | 2.1 | 0.1 | 11.1 |
|  | Salmonella sepsis (10058878) | 3 | 5.9 | 1.2 | 16.2 |  | 7 | 13.0 | 5.4 | 24.9 |  | 3 | 6.3 | 1.3 | 17.2 |
|  | Sepsis (10040047) | 8 | 15.7 | 7.0 | 28.6 |  | 1 | 1.9 | 0.0 | 9.9 |  | 4 | 8.3 | 2.3 | 20.0 |
|  | Skin bacterial infection (10052891) | 1 | 2.0 | 0.0 | 10.4 |  | 0 | 0.0 | 0.0 | 6.6 |  | 0 | 0.0 | 0.0 | 7.4 |
|  | Staphylococcal sepsis (10056430) | 0 | 0.0 | 0.0 | 7.0 |  | 1 | 1.9 | 0.0 | 9.9 |  | 0 | 0.0 | 0.0 | 7.4 |
|  | Streptococcal sepsis (10048960) | 0 | 0.0 | 0.0 | 7.0 |  | 0 | 0.0 | 0.0 | 6.6 |  | 2 | 4.2 | 0.5 | 14.3 |
|  | Subcutaneous abscess (10042343) | 1 | 2.0 | 0.0 | 10.4 |  | 0 | 0.0 | 0.0 | 6.6 |  | 0 | 0.0 | 0.0 | 7.4 |
|  | Superinfection (10042566) | 0 | 0.0 | 0.0 | 7.0 |  | 1 | 1.9 | 0.0 | 9.9 |  | 0 | 0.0 | 0.0 | 7.4 |
|  | Tuberculosis (10044755) | 2 | 3.9 | 0.5 | 13.5 |  | 4 | 7.4 | 2.1 | 17.9 |  | 5 | 10.4 | 3.5 | 22.7 |
|  | Typhoid fever (10045275) | 0 | 0.0 | 0.0 | 7.0 |  | 0 | 0.0 | 0.0 | 6.6 |  | 1 | 2.1 | 0.1 | 11.1 |
|  | Upper respiratory tract infection (10046306) | 2 | 3.9 | 0.5 | 13.5 |  | 2 | 3.7 | 0.5 | 12.7 |  | 2 | 4.2 | 0.5 | 14.3 |
|  | Urinary tract infection (10046571) | 3 | 5.9 | 1.2 | 16.2 |  | 5 | 9.3 | 3.1 | 20.3 |  | 3 | 6.3 | 1.3 | 17.2 |
| Injury, poisoning and procedural complications (10022117) | Soft tissue injury (10041291) | 1 | 2.0 | 0.0 | 10.4 |  | 0 | 0.0 | 0.0 | 6.6 |  | 1 | 2.1 | 0.1 | 11.1 |
|  | Thermal burn (10053615) | 1 | 2.0 | 0.0 | 10.4 |  | 0 | 0.0 | 0.0 | 6.6 |  | 1 | 2.1 | 0.1 | 11.1 |
|  | Wrist fracture (10048049) | 0 | 0.0 | 0.0 | 7.0 |  | 0 | 0.0 | 0.0 | 6.6 |  | 1 | 2.1 | 0.1 | 11.1 |
| Metabolism and nutrition disorders (10027433) | Failure to thrive (10016165) | 0 | 0.0 | 0.0 | 7.0 |  | 0 | 0.0 | 0.0 | 6.6 |  | 1 | 2.1 | 0.1 | 11.1 |
|  | Hypoglycemia (10020993) | 1 | 2.0 | 0.0 | 10.4 |  | 1 | 1.9 | 0.0 | 9.9 |  | 0 | 0.0 | 0.0 | 7.4 |
|  | Hypokalemia (10021015) | 0 | 0.0 | 0.0 | 7.0 |  | 1 | 1.9 | 0.0 | 9.9 |  | 0 | 0.0 | 0.0 | 7.4 |
|  | Kwashiorkor (10023504) | 5 | 9.8 | 3.3 | 21.4 |  | 3 | 5.6 | 1.2 | 15.4 |  | 3 | 6.3 | 1.3 | 17.2 |
|  | Malnutrition (10061273) | 6 | 11.8 | 4.4 | 23.9 |  | 11 | 20.4 | 10.6 | 33.5 |  | 8 | 16.7 | 7.5 | 30.2 |
|  | Marasmus (10026820) | 5 | 9.8 | 3.3 | 21.4 |  | 5 | 9.3 | 3.1 | 20.3 |  | 4 | 8.3 | 2.3 | 20.0 |
|  | Underweight (10048828) | 0 | 0.0 | 0.0 | 7.0 |  | 1 | 1.9 | 0.0 | 9.9 |  | 0 | 0.0 | 0.0 | 7.4 |
| Musculoskeletal and connective tissue disorders (10028395) | Arthritis (10003246) | 1 | 2.0 | 0.0 | 10.4 |  | 1 | 1.9 | 0.0 | 9.9 |  | 0 | 0.0 | 0.0 | 7.4 |
| Nervous system disorders (10029205) | Convulsion (10010904) | 4 | 7.8 | 2.2 | 18.9 |  | 2 | 3.7 | 0.5 | 12.7 |  | 4 | 8.3 | 2.3 | 20.0 |
|  | Epilepsy (10015037) | 0 | 0.0 | 0.0 | 7.0 |  | 2 | 3.7 | 0.5 | 12.7 |  | 0 | 0.0 | 0.0 | 7.4 |
|  | Febrile convulsion (10016284) | 6 | 11.8 | 4.4 | 23.9 |  | 5 | 9.3 | 3.1 | 20.3 |  | 3 | 6.3 | 1.3 | 17.2 |
|  | Metabolic encephalopathy (10062190) | 0 | 0.0 | 0.0 | 7.0 |  | 1 | 1.9 | 0.0 | 9.9 |  | 0 | 0.0 | 0.0 | 7.4 |
|  | Uremic encephalopathy (10046326) | 0 | 0.0 | 0.0 | 7.0 |  | 0 | 0.0 | 0.0 | 6.6 |  | 1 | 2.1 | 0.1 | 11.1 |
| Respiratory, thoracic and mediastinal disorders (10038738) | Interstitial lung disease (10022611) | 0 | 0.0 | 0.0 | 7.0 |  | 0 | 0.0 | 0.0 | 6.6 |  | 1 | 2.1 | 0.1 | 11.1 |
|  | Pleural effusion (10035598) | 1 | 2.0 | 0.0 | 10.4 |  | 0 | 0.0 | 0.0 | 6.6 |  | 0 | 0.0 | 0.0 | 7.4 |
| Skin and subcutaneous tissue disorders (10040785) | Dermatitis (10012431) | 0 | 0.0 | 0.0 | 7.0 |  | 0 | 0.0 | 0.0 | 6.6 |  | 1 | 2.1 | 0.1 | 11.1 |
|  | Rash maculo-papular (10037868) | 1 | 2.0 | 0.0 | 10.4 |  | 0 | 0.0 | 0.0 | 6.6 |  | 0 | 0.0 | 0.0 | 7.4 |
|  | Stevens-Johnson syndrome (10042033) | 0 | 0.0 | 0.0 | 7.0 |  | 0 | 0.0 | 0.0 | 6.6 |  | 1 | 2.1 | 0.1 | 11.1 |
| Vascular disorders (10047065) | Hypovolemic shock (10021138) | 0 | 0.0 | 0.0 | 7.0 |  | 1 | 1.9 | 0.0 | 9.9 |  | 0 | 0.0 | 0.0 | 7.4 |
|  | Shock (10040560) | 1 | 2.0 | 0.0 | 10.4 |  | 1 | 1.9 | 0.0 | 9.9 |  | 0 | 0.0 | 0.0 | 7.4 |

ITT, intent-to-treat; R3R, group receiving 4 doses of RTS,S/AS01; R3C, group receiving 3 doses of RTS,S/AS01 plus 1 dose of comparator vaccine; C3C, group receiving 4 doses of comparator vaccine; N, number of participants with at least one administered vaccine dose; n (%), number (percentage) of participants reporting the event at least once; CI, confidence interval; LL, lower limit; UL, upper limit; SAE, serious adverse event.

^a^While all children included in these analyses were confirmed to be HIV-infected, not all HIV infections were reported as SAEs; the decision to report an HIV infection as an SAE was based on the investigators’ judgment of whether it met the criteria of an SAE.

**Supplementary table 5. Number and percentage of HIV-infected participants in the 5–17 months age category for whom serious adverse events were reported between dose 1 and study end, listed by Medical Dictionary for Regulatory Activities system organ class and preferred term (ITT population of HIV-infected participants)**

|  | | **R3R  N=24** | | | |  | **R3C  N=29** | | | |  | **C3C  N=32** | | | |
| --- | --- | --- | --- | --- | --- | --- | --- | --- | --- | --- | --- | --- | --- | --- | --- |
|  | |  | | **95% CI** | |  |  | | **95% CI** | |  |  | | **95% CI** | |
| **Primary System Organ Class (CODE)** | **Preferred Term (CODE)** | **n** | **%** | **LL** | **UL** |  | **n** | **%** | **LL** | **UL** |  | **n** | **%** | **LL** | **UL** |
| At least one SAE |  | 22 | 91.7 | 73.0 | 99.0 |  | 24 | 82.8 | 64.2 | 94.2 |  | 27 | 84.4 | 67.2 | 94.7 |
| Blood and lymphatic system disorders (10005329) | Anemia (10002034) | 5 | 20.8 | 7.1 | 42.2 |  | 7 | 24.1 | 10.3 | 43.5 |  | 9 | 28.1 | 13.7 | 46.7 |
|  | Disseminated intravascular coagulation (10013442) | 1 | 4.2 | 0.1 | 21.1 |  | 0 | 0.0 | 0.0 | 11.9 |  | 0 | 0.0 | 0.0 | 10.9 |
|  | Neutropenia (10029354) | 0 | 0.0 | 0.0 | 14.2 |  | 1 | 3.4 | 0.1 | 17.8 |  | 0 | 0.0 | 0.0 | 10.9 |
| Gastrointestinal disorders (10017947) | Colitis (10009887) | 0 | 0.0 | 0.0 | 14.2 |  | 0 | 0.0 | 0.0 | 11.9 |  | 1 | 3.1 | 0.1 | 16.2 |
|  | Enteritis (10014866) | 0 | 0.0 | 0.0 | 14.2 |  | 1 | 3.4 | 0.1 | 17.8 |  | 1 | 3.1 | 0.1 | 16.2 |
| General disorders and administration site conditions (10018065) | Hypothermia (10021113) | 1 | 4.2 | 0.1 | 21.1 |  | 0 | 0.0 | 0.0 | 11.9 |  | 0 | 0.0 | 0.0 | 10.9 |
|  | Pyrexia (10037660) | 1 | 4.2 | 0.1 | 21.1 |  | 1 | 3.4 | 0.1 | 17.8 |  | 1 | 3.1 | 0.1 | 16.2 |
| Hepatobiliary disorders (10019805) | Hepatitis acute (10019727) | 0 | 0.0 | 0.0 | 14.2 |  | 1 | 3.4 | 0.1 | 17.8 |  | 0 | 0.0 | 0.0 | 10.9 |
| Immune system disorders (10021428) | Anaphylactic reaction (10002198) | 0 | 0.0 | 0.0 | 14.2 |  | 0 | 0.0 | 0.0 | 11.9 |  | 1 | 3.1 | 0.1 | 16.2 |
| Infections and infestations (10021881) | Aids dementia complex (10065040) | 0 | 0.0 | 0.0 | 14.2 |  | 0 | 0.0 | 0.0 | 11.9 |  | 1 | 3.1 | 0.1 | 16.2 |
|  | Bronchiolitis (10006448) | 1 | 4.2 | 0.1 | 21.1 |  | 0 | 0.0 | 0.0 | 11.9 |  | 0 | 0.0 | 0.0 | 10.9 |
|  | Bronchitis (10006451) | 0 | 0.0 | 0.0 | 14.2 |  | 0 | 0.0 | 0.0 | 11.9 |  | 1 | 3.1 | 0.1 | 16.2 |
|  | Bronchopneumonia (10006469) | 2 | 8.3 | 1.0 | 27.0 |  | 4 | 13.8 | 3.9 | 31.7 |  | 2 | 6.3 | 0.8 | 20.8 |
|  | Cellulitis of male external genital organ (10064929) | 1 | 4.2 | 0.1 | 21.1 |  | 0 | 0.0 | 0.0 | 11.9 |  | 0 | 0.0 | 0.0 | 10.9 |
|  | Conjunctivitis (10010741) | 1 | 4.2 | 0.1 | 21.1 |  | 0 | 0.0 | 0.0 | 11.9 |  | 0 | 0.0 | 0.0 | 10.9 |
|  | Disseminated tuberculosis (10013453) | 1 | 4.2 | 0.1 | 21.1 |  | 0 | 0.0 | 0.0 | 11.9 |  | 1 | 3.1 | 0.1 | 16.2 |
|  | Dysentery (10051402) | 0 | 0.0 | 0.0 | 14.2 |  | 1 | 3.4 | 0.1 | 17.8 |  | 0 | 0.0 | 0.0 | 10.9 |
|  | Encephalitis (10014581) | 0 | 0.0 | 0.0 | 14.2 |  | 0 | 0.0 | 0.0 | 11.9 |  | 1 | 3.1 | 0.1 | 16.2 |
|  | Enterococcal sepsis (10054221) | 1 | 4.2 | 0.1 | 21.1 |  | 0 | 0.0 | 0.0 | 11.9 |  | 0 | 0.0 | 0.0 | 10.9 |
|  | Gastroenteritis (10017888) | 9 | 37.5 | 18.8 | 59.4 |  | 12 | 41.4 | 23.5 | 61.1 |  | 13 | 40.6 | 23.7 | 59.4 |
|  | Gastroenteritis salmonella (10017914) | 1 | 4.2 | 0.1 | 21.1 |  | 0 | 0.0 | 0.0 | 11.9 |  | 0 | 0.0 | 0.0 | 10.9 |
|  | Gastroenteritis shigella (10017915) | 0 | 0.0 | 0.0 | 14.2 |  | 0 | 0.0 | 0.0 | 11.9 |  | 1 | 3.1 | 0.1 | 16.2 |
|  | Gastrointestinal candidiasis (10017938) | 0 | 0.0 | 0.0 | 14.2 |  | 1 | 3.4 | 0.1 | 17.8 |  | 0 | 0.0 | 0.0 | 10.9 |
|  | Groin abscess (10050269) | 1 | 4.2 | 0.1 | 21.1 |  | 0 | 0.0 | 0.0 | 11.9 |  | 0 | 0.0 | 0.0 | 10.9 |
|  | Helminthic infection (10061201) | 0 | 0.0 | 0.0 | 14.2 |  | 1 | 3.4 | 0.1 | 17.8 |  | 2 | 6.3 | 0.8 | 20.8 |
|  | HIV infection (10020161)^a^ | 19 | 79.2 | 57.8 | 92.9 |  | 19 | 65.5 | 45.7 | 82.1 |  | 16 | 50.0 | 31.9 | 68.1 |
|  | HIV infection who clinical stage ii (10064447)^a^ | 0 | 0.0 | 0.0 | 14.2 |  | 0 | 0.0 | 0.0 | 11.9 |  | 1 | 3.1 | 0.1 | 16.2 |
|  | HIV infection who clinical stage iv (10064456)^a^ | 0 | 0.0 | 0.0 | 14.2 |  | 1 | 3.4 | 0.1 | 17.8 |  | 0 | 0.0 | 0.0 | 10.9 |
|  | Impetigo (10021531) | 0 | 0.0 | 0.0 | 14.2 |  | 0 | 0.0 | 0.0 | 11.9 |  | 1 | 3.1 | 0.1 | 16.2 |
|  | Infected skin ulcer (10021784) | 0 | 0.0 | 0.0 | 14.2 |  | 1 | 3.4 | 0.1 | 17.8 |  | 0 | 0.0 | 0.0 | 10.9 |
|  | Klebsiella sepsis (10054160) | 1 | 4.2 | 0.1 | 21.1 |  | 0 | 0.0 | 0.0 | 11.9 |  | 0 | 0.0 | 0.0 | 10.9 |
|  | Lobar pneumonia (10024738) | 3 | 12.5 | 2.7 | 32.4 |  | 2 | 6.9 | 0.8 | 22.8 |  | 4 | 12.5 | 3.5 | 29.0 |
|  | Lymph node abscess (10050167) | 0 | 0.0 | 0.0 | 14.2 |  | 1 | 3.4 | 0.1 | 17.8 |  | 0 | 0.0 | 0.0 | 10.9 |
|  | Lymph node tuberculosis (10025183) | 0 | 0.0 | 0.0 | 14.2 |  | 0 | 0.0 | 0.0 | 11.9 |  | 1 | 3.1 | 0.1 | 16.2 |
|  | Malaria (10025487) | 7 | 29.2 | 12.6 | 51.1 |  | 11 | 37.9 | 20.7 | 57.7 |  | 10 | 31.3 | 16.1 | 50.0 |
|  | Meningitis (10027199) | 2 | 8.3 | 1.0 | 27.0 |  | 0 | 0.0 | 0.0 | 11.9 |  | 1 | 3.1 | 0.1 | 16.2 |
|  | Oral candidiasis (10030963) | 3 | 12.5 | 2.7 | 32.4 |  | 2 | 6.9 | 0.8 | 22.8 |  | 2 | 6.3 | 0.8 | 20.8 |
|  | Oropharyngeal candidiasis (10050346) | 1 | 4.2 | 0.1 | 21.1 |  | 1 | 3.4 | 0.1 | 17.8 |  | 0 | 0.0 | 0.0 | 10.9 |
|  | Otitis media (10033078) | 1 | 4.2 | 0.1 | 21.1 |  | 2 | 6.9 | 0.8 | 22.8 |  | 4 | 12.5 | 3.5 | 29.0 |
|  | Parotitis (10034038) | 0 | 0.0 | 0.0 | 14.2 |  | 1 | 3.4 | 0.1 | 17.8 |  | 0 | 0.0 | 0.0 | 10.9 |
|  | Pharyngitis (10034835) | 1 | 4.2 | 0.1 | 21.1 |  | 0 | 0.0 | 0.0 | 11.9 |  | 0 | 0.0 | 0.0 | 10.9 |
|  | Pneumococcal sepsis (10054047) | 1 | 4.2 | 0.1 | 21.1 |  | 1 | 3.4 | 0.1 | 17.8 |  | 0 | 0.0 | 0.0 | 10.9 |
|  | Pneumocystis jirovecii pneumonia (10073755) | 2 | 8.3 | 1.0 | 27.0 |  | 0 | 0.0 | 0.0 | 11.9 |  | 1 | 3.1 | 0.1 | 16.2 |
|  | Pneumonia (10035664) | 7 | 29.2 | 12.6 | 51.1 |  | 12 | 41.4 | 23.5 | 61.1 |  | 15 | 46.9 | 29.1 | 65.3 |
|  | Pulmonary tuberculosis (10037440) | 4 | 16.7 | 4.7 | 37.4 |  | 0 | 0.0 | 0.0 | 11.9 |  | 0 | 0.0 | 0.0 | 10.9 |
|  | Pyoderma (10037632) | 0 | 0.0 | 0.0 | 14.2 |  | 0 | 0.0 | 0.0 | 11.9 |  | 1 | 3.1 | 0.1 | 16.2 |
|  | Salmonella sepsis (10058878) | 2 | 8.3 | 1.0 | 27.0 |  | 3 | 10.3 | 2.2 | 27.4 |  | 2 | 6.3 | 0.8 | 20.8 |
|  | Sepsis (10040047) | 4 | 16.7 | 4.7 | 37.4 |  | 1 | 3.4 | 0.1 | 17.8 |  | 2 | 6.3 | 0.8 | 20.8 |
|  | Skin bacterial infection (10052891) | 1 | 4.2 | 0.1 | 21.1 |  | 0 | 0.0 | 0.0 | 11.9 |  | 0 | 0.0 | 0.0 | 10.9 |
|  | Streptococcal sepsis (10048960) | 0 | 0.0 | 0.0 | 14.2 |  | 0 | 0.0 | 0.0 | 11.9 |  | 1 | 3.1 | 0.1 | 16.2 |
|  | Subcutaneous abscess (10042343) | 1 | 4.2 | 0.1 | 21.1 |  | 0 | 0.0 | 0.0 | 11.9 |  | 0 | 0.0 | 0.0 | 10.9 |
|  | Tuberculosis (10044755) | 1 | 4.2 | 0.1 | 21.1 |  | 2 | 6.9 | 0.8 | 22.8 |  | 3 | 9.4 | 2.0 | 25.0 |
|  | Typhoid fever (10045275) | 0 | 0.0 | 0.0 | 14.2 |  | 0 | 0.0 | 0.0 | 11.9 |  | 1 | 3.1 | 0.1 | 16.2 |
|  | Upper respiratory tract infection (10046306) | 2 | 8.3 | 1.0 | 27.0 |  | 0 | 0.0 | 0.0 | 11.9 |  | 2 | 6.3 | 0.8 | 20.8 |
|  | Urinary tract infection (10046571) | 0 | 0.0 | 0.0 | 14.2 |  | 3 | 10.3 | 2.2 | 27.4 |  | 3 | 9.4 | 2.0 | 25.0 |
| Injury, poisoning and procedural complications (10022117) | Thermal burn (10053615) | 0 | 0.0 | 0.0 | 14.2 |  | 0 | 0.0 | 0.0 | 11.9 |  | 1 | 3.1 | 0.1 | 16.2 |
| Metabolism and nutrition disorders (10027433) | Failure to thrive (10016165) | 0 | 0.0 | 0.0 | 14.2 |  | 0 | 0.0 | 0.0 | 11.9 |  | 1 | 3.1 | 0.1 | 16.2 |
|  | Hypoglycaemia (10020993) | 1 | 4.2 | 0.1 | 21.1 |  | 1 | 3.4 | 0.1 | 17.8 |  | 0 | 0.0 | 0.0 | 10.9 |
|  | Hypokalaemia (10021015) | 0 | 0.0 | 0.0 | 14.2 |  | 1 | 3.4 | 0.1 | 17.8 |  | 0 | 0.0 | 0.0 | 10.9 |
|  | Kwashiorkor (10023504) | 4 | 16.7 | 4.7 | 37.4 |  | 1 | 3.4 | 0.1 | 17.8 |  | 2 | 6.3 | 0.8 | 20.8 |
|  | Malnutrition (10061273) | 2 | 8.3 | 1.0 | 27.0 |  | 7 | 24.1 | 10.3 | 43.5 |  | 5 | 15.6 | 5.3 | 32.8 |
|  | Marasmus (10026820) | 1 | 4.2 | 0.1 | 21.1 |  | 2 | 6.9 | 0.8 | 22.8 |  | 1 | 3.1 | 0.1 | 16.2 |
|  | Underweight (10048828) | 0 | 0.0 | 0.0 | 14.2 |  | 1 | 3.4 | 0.1 | 17.8 |  | 0 | 0.0 | 0.0 | 10.9 |
| Musculoskeletal and connective tissue disorders (10028395) | Arthritis (10003246) | 1 | 4.2 | 0.1 | 21.1 |  | 0 | 0.0 | 0.0 | 11.9 |  | 0 | 0.0 | 0.0 | 10.9 |
| Nervous system disorders (10029205) | Convulsion (10010904) | 4 | 16.7 | 4.7 | 37.4 |  | 2 | 6.9 | 0.8 | 22.8 |  | 4 | 12.5 | 3.5 | 29.0 |
|  | Epilepsy (10015037) | 0 | 0.0 | 0.0 | 14.2 |  | 2 | 6.9 | 0.8 | 22.8 |  | 0 | 0.0 | 0.0 | 10.9 |
|  | Febrile convulsion (10016284) | 4 | 16.7 | 4.7 | 37.4 |  | 3 | 10.3 | 2.2 | 27.4 |  | 3 | 9.4 | 2.0 | 25.0 |
| Respiratory, thoracic and mediastinal disorders (10038738) | Interstitial lung disease (10022611) | 0 | 0.0 | 0.0 | 14.2 |  | 0 | 0.0 | 0.0 | 11.9 |  | 1 | 3.1 | 0.1 | 16.2 |
| Skin and subcutaneous tissue disorders (10040785) | Dermatitis (10012431) | 0 | 0.0 | 0.0 | 14.2 |  | 0 | 0.0 | 0.0 | 11.9 |  | 1 | 3.1 | 0.1 | 16.2 |
|  | Rash maculo-papular (10037868) | 1 | 4.2 | 0.1 | 21.1 |  | 0 | 0.0 | 0.0 | 11.9 |  | 0 | 0.0 | 0.0 | 10.9 |
|  | Stevens-Johnson syndrome (10042033) | 0 | 0.0 | 0.0 | 14.2 |  | 0 | 0.0 | 0.0 | 11.9 |  | 1 | 3.1 | 0.1 | 16.2 |

ITT, intent-to-treat; R3R, group receiving 4 doses of RTS,S/AS01; R3C, group receiving 3 doses of RTS,S/AS01 plus 1 dose of comparator vaccine; C3C, group receiving 4 doses of comparator vaccine; N, number of participants with at least one administered vaccine dose; n (%), number (percentage) of participants reporting the event at least once; CI, confidence interval; LL, lower limit; UL, upper limit; SAE, serious adverse event.

^a^While all children included in these analyses were confirmed to be HIV-infected, not all HIV infections were reported as SAEs; the decision to report an HIV infection as an SAE was based on the investigators’ judgment of whether it met the criteria of an SAE.

**Supplementary table 6. Number and percentage of HIV-infected participants in the 6–12 weeks age category for whom serious adverse events were reported between dose 1 and study end, listed by Medical Dictionary for Regulatory Activities system organ class and preferred term (ITT population of HIV-infected participants)**

|  | | **R3R  N = 27** | | | |  | **R3C  N = 25** | | | |  | **C3C  N = 16** | | | |
| --- | --- | --- | --- | --- | --- | --- | --- | --- | --- | --- | --- | --- | --- | --- | --- |
|  | |  | | **95% CI** | |  |  | | **95% CI** | |  |  | | **95% CI** | |
| **Primary System Organ Class (CODE)** | **Preferred Term (CODE)** | **n** | **%** | **LL** | **UL** |  | **n** | **%** | **LL** | **UL** |  | **n** | **%** | **LL** | **UL** |
| At least one SAE |  | 25 | 92.6 | 75.7 | 99.1 |  | 22 | 88.0 | 68.8 | 97.5 |  | 15 | 93.8 | 69.8 | 99.8 |
| Blood and lymphatic system disorders (10005329) | Anemia (10002034) | 4 | 14.8 | 4.2 | 33.7 |  | 4 | 16.0 | 4.5 | 36.1 |  | 5 | 31.3 | 11.0 | 58.7 |
| Cardiac disorders (10007541) | Pericardial effusion (10034474) | 0 | 0.0 | 0.0 | 12.8 |  | 0 | 0.0 | 0.0 | 13.7 |  | 1 | 6.3 | 0.2 | 30.2 |
| Congenital, familial and genetic disorders (10010331) | Sickle cell anemia with crisis (10040642) | 0 | 0.0 | 0.0 | 12.8 |  | 1 | 4.0 | 0.1 | 20.4 |  | 0 | 0.0 | 0.0 | 20.6 |
| Gastrointestinal disorders (10017947) | Enteritis (10014866) | 0 | 0.0 | 0.0 | 12.8 |  | 0 | 0.0 | 0.0 | 13.7 |  | 1 | 6.3 | 0.2 | 30.2 |
|  | Vomiting (10047700) | 1 | 3.7 | 0.1 | 19.0 |  | 0 | 0.0 | 0.0 | 13.7 |  | 0 | 0.0 | 0.0 | 20.6 |
| Immune system disorders (10021428) | Immune reconstitution inflammatory syndrome (10065042) | 0 | 0.0 | 0.0 | 12.8 |  | 0 | 0.0 | 0.0 | 13.7 |  | 1 | 6.3 | 0.2 | 30.2 |
| Infections and infestations (10021881) | Abscess (10000269) | 0 | 0.0 | 0.0 | 12.8 |  | 1 | 4.0 | 0.1 | 20.4 |  | 0 | 0.0 | 0.0 | 20.6 |
|  | Abscess limb (10050473) | 0 | 0.0 | 0.0 | 12.8 |  | 0 | 0.0 | 0.0 | 13.7 |  | 1 | 6.3 | 0.2 | 30.2 |
|  | Arthritis bacterial (10053555) | 0 | 0.0 | 0.0 | 12.8 |  | 1 | 4.0 | 0.1 | 20.4 |  | 0 | 0.0 | 0.0 | 20.6 |
|  | Bronchopneumonia (10006469) | 1 | 3.7 | 0.1 | 19.0 |  | 3 | 12.0 | 2.5 | 31.2 |  | 2 | 12.5 | 1.6 | 38.3 |
|  | Conjunctivitis (10010741) | 0 | 0.0 | 0.0 | 12.8 |  | 1 | 4.0 | 0.1 | 20.4 |  | 0 | 0.0 | 0.0 | 20.6 |
|  | Dysentery (10051402) | 0 | 0.0 | 0.0 | 12.8 |  | 1 | 4.0 | 0.1 | 20.4 |  | 0 | 0.0 | 0.0 | 20.6 |
|  | Encephalitis (10014581) | 1 | 3.7 | 0.1 | 19.0 |  | 0 | 0.0 | 0.0 | 13.7 |  | 0 | 0.0 | 0.0 | 20.6 |
|  | Escherichia sepsis (10015296) | 1 | 3.7 | 0.1 | 19.0 |  | 0 | 0.0 | 0.0 | 13.7 |  | 0 | 0.0 | 0.0 | 20.6 |
|  | Gastroenteritis (10017888) | 13 | 48.1 | 28.7 | 68.1 |  | 11 | 44.0 | 24.4 | 65.1 |  | 4 | 25.0 | 7.3 | 52.4 |
|  | Gastroenteritis salmonella (10017914) | 0 | 0.0 | 0.0 | 12.8 |  | 1 | 4.0 | 0.1 | 20.4 |  | 0 | 0.0 | 0.0 | 20.6 |
|  | HIV associated nephropathy (10070737) | 0 | 0.0 | 0.0 | 12.8 |  | 0 | 0.0 | 0.0 | 13.7 |  | 1 | 6.3 | 0.2 | 30.2 |
|  | HIV infection (10020161)^a^ | 19 | 70.4 | 49.8 | 86.2 |  | 15 | 60.0 | 38.7 | 78.9 |  | 10 | 62.5 | 35.4 | 84.8 |
|  | HIV infection who clinical stage iii (10064448)^a^ | 1 | 3.7 | 0.1 | 19.0 |  | 1 | 4.0 | 0.1 | 20.4 |  | 0 | 0.0 | 0.0 | 20.6 |
|  | HIV infection who clinical stage iv (10064456)^a^ | 1 | 3.7 | 0.1 | 19.0 |  | 0 | 0.0 | 0.0 | 13.7 |  | 0 | 0.0 | 0.0 | 20.6 |
|  | Impetigo (10021531) | 0 | 0.0 | 0.0 | 12.8 |  | 0 | 0.0 | 0.0 | 13.7 |  | 1 | 6.3 | 0.2 | 30.2 |
|  | Lobar pneumonia (10024738) | 5 | 18.5 | 6.3 | 38.1 |  | 2 | 8.0 | 1.0 | 26.0 |  | 3 | 18.8 | 4.0 | 45.6 |
|  | Malaria (10025487) | 5 | 18.5 | 6.3 | 38.1 |  | 7 | 28.0 | 12.1 | 49.4 |  | 1 | 6.3 | 0.2 | 30.2 |
|  | Meningitis pneumococcal (10027253) | 0 | 0.0 | 0.0 | 12.8 |  | 1 | 4.0 | 0.1 | 20.4 |  | 0 | 0.0 | 0.0 | 20.6 |
|  | Oral candidiasis (10030963) | 0 | 0.0 | 0.0 | 12.8 |  | 1 | 4.0 | 0.1 | 20.4 |  | 0 | 0.0 | 0.0 | 20.6 |
|  | Osteomyelitis (10031252) | 0 | 0.0 | 0.0 | 12.8 |  | 0 | 0.0 | 0.0 | 13.7 |  | 1 | 6.3 | 0.2 | 30.2 |
|  | Otitis media (10033078) | 1 | 3.7 | 0.1 | 19.0 |  | 3 | 12.0 | 2.5 | 31.2 |  | 1 | 6.3 | 0.2 | 30.2 |
|  | Parotitis (10034038) | 0 | 0.0 | 0.0 | 12.8 |  | 1 | 4.0 | 0.1 | 20.4 |  | 0 | 0.0 | 0.0 | 20.6 |
|  | Periorbital cellulitis (10057182) | 0 | 0.0 | 0.0 | 12.8 |  | 0 | 0.0 | 0.0 | 13.7 |  | 1 | 6.3 | 0.2 | 30.2 |
|  | Pneumococcal sepsis (10054047) | 1 | 3.7 | 0.1 | 19.0 |  | 2 | 8.0 | 1.0 | 26.0 |  | 0 | 0.0 | 0.0 | 20.6 |
|  | Pneumocystis jirovecii pneumonia (10073755) | 3 | 11.1 | 2.4 | 29.2 |  | 1 | 4.0 | 0.1 | 20.4 |  | 0 | 0.0 | 0.0 | 20.6 |
|  | Pneumonia (10035664) | 11 | 40.7 | 22.4 | 61.2 |  | 13 | 52.0 | 31.3 | 72.2 |  | 6 | 37.5 | 15.2 | 64.6 |
|  | Pneumonia pneumococcal (10035728) | 0 | 0.0 | 0.0 | 12.8 |  | 1 | 4.0 | 0.1 | 20.4 |  | 0 | 0.0 | 0.0 | 20.6 |
|  | Pneumonia viral (10035737) | 0 | 0.0 | 0.0 | 12.8 |  | 1 | 4.0 | 0.1 | 20.4 |  | 0 | 0.0 | 0.0 | 20.6 |
|  | Pulmonary tuberculosis (10037440) | 4 | 14.8 | 4.2 | 33.7 |  | 4 | 16.0 | 4.5 | 36.1 |  | 0 | 0.0 | 0.0 | 20.6 |
|  | Salmonella sepsis (10058878) | 1 | 3.7 | 0.1 | 19.0 |  | 4 | 16.0 | 4.5 | 36.1 |  | 1 | 6.3 | 0.2 | 30.2 |
|  | Sepsis (10040047) | 4 | 14.8 | 4.2 | 33.7 |  | 0 | 0.0 | 0.0 | 13.7 |  | 2 | 12.5 | 1.6 | 38.3 |
|  | Staphylococcal sepsis (10056430) | 0 | 0.0 | 0.0 | 12.8 |  | 1 | 4.0 | 0.1 | 20.4 |  | 0 | 0.0 | 0.0 | 20.6 |
|  | Streptococcal sepsis (10048960) | 0 | 0.0 | 0.0 | 12.8 |  | 0 | 0.0 | 0.0 | 13.7 |  | 1 | 6.3 | 0.2 | 30.2 |
|  | Superinfection (10042566) | 0 | 0.0 | 0.0 | 12.8 |  | 1 | 4.0 | 0.1 | 20.4 |  | 0 | 0.0 | 0.0 | 20.6 |
|  | Tuberculosis (10044755) | 1 | 3.7 | 0.1 | 19.0 |  | 2 | 8.0 | 1.0 | 26.0 |  | 2 | 12.5 | 1.6 | 38.3 |
|  | Upper respiratory tract infection (10046306) | 0 | 0.0 | 0.0 | 12.8 |  | 2 | 8.0 | 1.0 | 26.0 |  | 0 | 0.0 | 0.0 | 20.6 |
|  | Urinary tract infection (10046571) | 3 | 11.1 | 2.4 | 29.2 |  | 2 | 8.0 | 1.0 | 26.0 |  | 0 | 0.0 | 0.0 | 20.6 |
| Injury, poisoning and procedural complications (10022117) | Soft tissue injury (10041291) | 1 | 3.7 | 0.1 | 19.0 |  | 0 | 0.0 | 0.0 | 13.7 |  | 1 | 6.3 | 0.2 | 30.2 |
|  | Thermal burn (10053615) | 1 | 3.7 | 0.1 | 19.0 |  | 0 | 0.0 | 0.0 | 13.7 |  | 0 | 0.0 | 0.0 | 20.6 |
|  | Wrist fracture (10048049) | 0 | 0.0 | 0.0 | 12.8 |  | 0 | 0.0 | 0.0 | 13.7 |  | 1 | 6.3 | 0.2 | 30.2 |
| Metabolism and nutrition disorders (10027433) | Kwashiorkor (10023504) | 1 | 3.7 | 0.1 | 19.0 |  | 2 | 8.0 | 1.0 | 26.0 |  | 1 | 6.3 | 0.2 | 30.2 |
|  | Malnutrition (10061273) | 4 | 14.8 | 4.2 | 33.7 |  | 4 | 16.0 | 4.5 | 36.1 |  | 3 | 18.8 | 4.0 | 45.6 |
|  | Marasmus (10026820) | 4 | 14.8 | 4.2 | 33.7 |  | 3 | 12.0 | 2.5 | 31.2 |  | 3 | 18.8 | 4.0 | 45.6 |
| Musculoskeletal and connective tissue disorders (10028395) | Arthritis (10003246) | 0 | 0.0 | 0.0 | 12.8 |  | 1 | 4.0 | 0.1 | 20.4 |  | 0 | 0.0 | 0.0 | 20.6 |
| Nervous system disorders (10029205) | Febrile convulsion (10016284) | 2 | 7.4 | 0.9 | 24.3 |  | 2 | 8.0 | 1.0 | 26.0 |  | 0 | 0.0 | 0.0 | 20.6 |
|  | Metabolic encephalopathy (10062190) | 0 | 0.0 | 0.0 | 12.8 |  | 1 | 4.0 | 0.1 | 20.4 |  | 0 | 0.0 | 0.0 | 20.6 |
|  | Uremic encephalopathy (10046326) | 0 | 0.0 | 0.0 | 12.8 |  | 0 | 0.0 | 0.0 | 13.7 |  | 1 | 6.3 | 0.2 | 30.2 |
| Respiratory, thoracic and mediastinal disorders (10038738) | Pleural effusion (10035598) | 1 | 3.7 | 0.1 | 19.0 |  | 0 | 0.0 | 0.0 | 13.7 |  | 0 | 0.0 | 0.0 | 20.6 |
| Vascular disorders (10047065) | Hypovolemic shock (10021138) | 0 | 0.0 | 0.0 | 12.8 |  | 1 | 4.0 | 0.1 | 20.4 |  | 0 | 0.0 | 0.0 | 20.6 |
|  | Shock (10040560) | 1 | 3.7 | 0.1 | 19.0 |  | 1 | 4.0 | 0.1 | 20.4 |  | 0 | 0.0 | 0.0 | 20.6 |

ITT, intent-to-treat; R3R, group receiving 4 doses of RTS,S/AS01; R3C, group receiving 3 doses of RTS,S/AS01 plus 1 dose of comparator vaccine; C3C, group receiving 4 doses of comparator vaccine; N, number of participants with at least one administered vaccine dose; n (%), number (percentage) of participants reporting the event at least once; CI, confidence interval; LL, lower limit; UL, upper limit; SAE, serious adverse event.

^a^While all children included in these analyses were confirmed to be HIV-infected, not all HIV infections were reported as SAEs; the decision to report an HIV infection as an SAE was based on the investigators’ judgment of whether it met the criteria of an SAE.

**Supplementary table 7. Number and percentage of HIV-infected participants for whom fatal serious adverse events were reported between dose 1 and study end, listed by Medical Dictionary for Regulatory Activities system organ class and preferred term (ITT population of HIV-infected participants)**

|  | | **R3R  N = 51** | | | |  | **R3C  N = 54** | | | |  | **C3C  N = 48** | | | |
| --- | --- | --- | --- | --- | --- | --- | --- | --- | --- | --- | --- | --- | --- | --- | --- |
|  | |  | | **95% CI** | |  |  | | **95% CI** | |  |  | | **95% CI** | |
| **Primary System Organ Class (CODE)** | **Preferred Term (CODE)** | **n** | **%** | **LL** | **UL** |  | **n** | **%** | **LL** | **UL** |  | **n** | **%** | **LL** | **UL** |
| At least one fatal SAE |  | 15 | 29.4 | 17.5 | 43.8 |  | 15 | 27.8 | 16.5 | 41.6 |  | 15 | 31.3 | 18.7 | 46.3 |
| Blood and lymphatic system disorders (10005329) | Anemia (10002034) | 2 | 3.9 | 0.5 | 13.5 |  | 2 | 3.7 | 0.5 | 12.7 |  | 2 | 4.2 | 0.5 | 14.3 |
|  | Disseminated intravascular coagulation (10013442) | 1 | 2.0 | 0.0 | 10.4 |  | 0 | 0.0 | 0.0 | 6.6 |  | 0 | 0.0 | 0.0 | 7.4 |
|  | Neutropenia (10029354) | 0 | 0.0 | 0.0 | 7.0 |  | 1 | 1.9 | 0.0 | 9.9 |  | 0 | 0.0 | 0.0 | 7.4 |
| General disorders and administration site conditions (10018065) | Pyrexia (10037660) | 0 | 0.0 | 0.0 | 7.0 |  | 0 | 0.0 | 0.0 | 6.6 |  | 1 | 2.1 | 0.1 | 11.1 |
| Infections and infestations (10021881) | Bronchopneumonia (10006469) | 1 | 2.0 | 0.0 | 10.4 |  | 0 | 0.0 | 0.0 | 6.6 |  | 0 | 0.0 | 0.0 | 7.4 |
|  | Disseminated tuberculosis (10013453) | 0 | 0.0 | 0.0 | 7.0 |  | 0 | 0.0 | 0.0 | 6.6 |  | 1 | 2.1 | 0.1 | 11.1 |
|  | Dysentery (10051402) | 0 | 0.0 | 0.0 | 7.0 |  | 2 | 3.7 | 0.5 | 12.7 |  | 0 | 0.0 | 0.0 | 7.4 |
|  | Encephalitis (10014581) | 1 | 2.0 | 0.0 | 10.4 |  | 0 | 0.0 | 0.0 | 6.6 |  | 1 | 2.1 | 0.1 | 11.1 |
|  | Gastroenteritis (10017888) | 6 | 11.8 | 4.4 | 23.9 |  | 5 | 9.3 | 3.1 | 20.3 |  | 4 | 8.3 | 2.3 | 20.0 |
|  | HIV associated nephropathy (10070737) | 0 | 0.0 | 0.0 | 7.0 |  | 0 | 0.0 | 0.0 | 6.6 |  | 1 | 2.1 | 0.1 | 11.1 |
|  | HIV infection (10020161)^a^ | 7 | 13.7 | 5.7 | 26.3 |  | 8 | 14.8 | 6.6 | 27.1 |  | 8 | 16.7 | 7.5 | 30.2 |
|  | HIV infection who clinical stage iii (10064448)^a^ | 0 | 0.0 | 0.0 | 7.0 |  | 1 | 1.9 | 0.0 | 9.9 |  | 0 | 0.0 | 0.0 | 7.4 |
|  | HIV infection who clinical stage iv (10064456)^a^ | 1 | 2.0 | 0.0 | 10.4 |  | 0 | 0.0 | 0.0 | 6.6 |  | 0 | 0.0 | 0.0 | 7.4 |
|  | Klebsiella sepsis (10054160) | 1 | 2.0 | 0.0 | 10.4 |  | 0 | 0.0 | 0.0 | 6.6 |  | 0 | 0.0 | 0.0 | 7.4 |
|  | Lobar pneumonia (10024738) | 0 | 0.0 | 0.0 | 7.0 |  | 0 | 0.0 | 0.0 | 6.6 |  | 3 | 6.3 | 1.3 | 17.2 |
|  | Lymph node tuberculosis (10025183) | 0 | 0.0 | 0.0 | 7.0 |  | 0 | 0.0 | 0.0 | 6.6 |  | 1 | 2.1 | 0.1 | 11.1 |
|  | Malaria (10025487) | 1 | 2.0 | 0.0 | 10.4 |  | 0 | 0.0 | 0.0 | 6.6 |  | 1 | 2.1 | 0.1 | 11.1 |
|  | Meningitis (10027199) | 1 | 2.0 | 0.0 | 10.4 |  | 0 | 0.0 | 0.0 | 6.6 |  | 1 | 2.1 | 0.1 | 11.1 |
|  | Oral candidiasis (10030963) | 0 | 0.0 | 0.0 | 7.0 |  | 0 | 0.0 | 0.0 | 6.6 |  | 2 | 4.2 | 0.5 | 14.3 |
|  | Oropharyngeal candidiasis (10050346) | 1 | 2.0 | 0.0 | 10.4 |  | 0 | 0.0 | 0.0 | 6.6 |  | 0 | 0.0 | 0.0 | 7.4 |
|  | Otitis media (10033078) | 1 | 2.0 | 0.0 | 10.4 |  | 1 | 1.9 | 0.0 | 9.9 |  | 1 | 2.1 | 0.1 | 11.1 |
|  | Pneumocystis jirovecii pneumonia (10073755) | 2 | 3.9 | 0.5 | 13.5 |  | 1 | 1.9 | 0.0 | 9.9 |  | 1 | 2.1 | 0.1 | 11.1 |
|  | Pneumonia (10035664) | 6 | 11.8 | 4.4 | 23.9 |  | 4 | 7.4 | 2.1 | 17.9 |  | 5 | 10.4 | 3.5 | 22.7 |
|  | Pulmonary tuberculosis (10037440) | 1 | 2.0 | 0.0 | 10.4 |  | 0 | 0.0 | 0.0 | 6.6 |  | 0 | 0.0 | 0.0 | 7.4 |
|  | Salmonella sepsis (10058878) | 1 | 2.0 | 0.0 | 10.4 |  | 1 | 1.9 | 0.0 | 9.9 |  | 1 | 2.1 | 0.1 | 11.1 |
|  | Sepsis (10040047) | 4 | 7.8 | 2.2 | 18.9 |  | 0 | 0.0 | 0.0 | 6.6 |  | 3 | 6.3 | 1.3 | 17.2 |
|  | Tuberculosis (10044755) | 0 | 0.0 | 0.0 | 7.0 |  | 1 | 1.9 | 0.0 | 9.9 |  | 2 | 4.2 | 0.5 | 14.3 |
|  | Typhoid fever (10045275) | 0 | 0.0 | 0.0 | 7.0 |  | 0 | 0.0 | 0.0 | 6.6 |  | 1 | 2.1 | 0.1 | 11.1 |
|  | Urinary tract infection (10046571) | 0 | 0.0 | 0.0 | 7.0 |  | 1 | 1.9 | 0.0 | 9.9 |  | 1 | 2.1 | 0.1 | 11.1 |
| Metabolism and nutrition disorders (10027433) | Kwashiorkor (10023504) | 0 | 0.0 | 0.0 | 7.0 |  | 1 | 1.9 | 0.0 | 9.9 |  | 1 | 2.1 | 0.1 | 11.1 |
|  | Malnutrition (10061273) | 1 | 2.0 | 0.0 | 10.4 |  | 2 | 3.7 | 0.5 | 12.7 |  | 3 | 6.3 | 1.3 | 17.2 |
|  | Marasmus (10026820) | 2 | 3.9 | 0.5 | 13.5 |  | 3 | 5.6 | 1.2 | 15.4 |  | 1 | 2.1 | 0.1 | 11.1 |
| Nervous system disorders (10029205) | Convulsion (10010904) | 0 | 0.0 | 0.0 | 7.0 |  | 0 | 0.0 | 0.0 | 6.6 |  | 4 | 8.3 | 2.3 | 20.0 |
|  | Febrile convulsion (10016284) | 1 | 2.0 | 0.0 | 10.4 |  | 0 | 0.0 | 0.0 | 6.6 |  | 0 | 0.0 | 0.0 | 7.4 |
|  | Uraemic encephalopathy (10046326) | 0 | 0.0 | 0.0 | 7.0 |  | 0 | 0.0 | 0.0 | 6.6 |  | 1 | 2.1 | 0.1 | 11.1 |

ITT, intent-to-treat; R3R, group receiving 4 doses of RTS,S/AS01; R3C, group receiving 3 doses of RTS,S/AS01 plus 1 dose of comparator vaccine; C3C, group receiving 4 doses of comparator vaccine; N, number of participants with at least one administered vaccine dose; n (%), number (percentage) of participants reporting the event at least once; CI, confidence interval; LL, lower limit; UL, upper limit; SAE, serious adverse event.

^a^While all children included in these analyses were confirmed to be HIV-infected, not all HIV infections were reported as SAEs; the decision to report an HIV infection as an SAE was based on the investigators’ judgment of whether it met the criteria of an SAE.

**Supplementary table 8. Number and percentage of HIV-infected participants for whom serious adverse events were reported during the 30-day periods after the first 3 doses, listed by Medical Dictionary for Regulatory Activities system organ class and preferred term (ITT population of HIV-infected participants)**

|  | | **R3R  N = 51** | | | |  | **R3C  N = 54** | | | |  | **C3C  N = 48** | | | |
| --- | --- | --- | --- | --- | --- | --- | --- | --- | --- | --- | --- | --- | --- | --- | --- |
|  | |  | | **95% CI** | |  |  | | **95% CI** | |  |  | | **95% CI** | |
| **Primary System Organ Class (CODE)** | **Preferred Term (CODE)** | **n** | **%** | **LL** | **UL** |  | **n** | **%** | **LL** | **UL** |  | **n** | **%** | **LL** | **UL** |
| At least one SAE |  | 14 | 27.5 | 15.9 | 41.7 |  | 16 | 29.6 | 18.0 | 43.6 |  | 12 | 25.0 | 13.6 | 39.6 |
| Blood and lymphatic system disorders (10005329) | Anaemia (10002034) | 1 | 2.0 | 0.0 | 10.4 |  | 0 | 0.0 | 0.0 | 6.6 |  | 2 | 4.2 | 0.5 | 14.3 |
|  | Disseminated intravascular coagulation (10013442) | 1 | 2.0 | 0.0 | 10.4 |  | 0 | 0.0 | 0.0 | 6.6 |  | 0 | 0.0 | 0.0 | 7.4 |
| Infections and infestations (10021881) | Arthritis bacterial (10053555) | 0 | 0.0 | 0.0 | 7.0 |  | 1 | 1.9 | 0.0 | 9.9 |  | 0 | 0.0 | 0.0 | 7.4 |
|  | Bronchitis (10006451) | 0 | 0.0 | 0.0 | 7.0 |  | 0 | 0.0 | 0.0 | 6.6 |  | 1 | 2.1 | 0.1 | 11.1 |
|  | Encephalitis (10014581) | 1 | 2.0 | 0.0 | 10.4 |  | 0 | 0.0 | 0.0 | 6.6 |  | 0 | 0.0 | 0.0 | 7.4 |
|  | Gastroenteritis (10017888) | 6 | 11.8 | 4.4 | 23.9 |  | 2 | 3.7 | 0.5 | 12.7 |  | 4 | 8.3 | 2.3 | 20.0 |
|  | HIV infection (10020161)^a^ | 5 | 9.8 | 3.3 | 21.4 |  | 8 | 14.8 | 6.6 | 27.1 |  | 5 | 10.4 | 3.5 | 22.7 |
|  | HIV infection who clinical stage ii (10064447)^a^ | 0 | 0.0 | 0.0 | 7.0 |  | 0 | 0.0 | 0.0 | 6.6 |  | 1 | 2.1 | 0.1 | 11.1 |
|  | HIV infection who clinical stage iii (10064448)^a^ | 0 | 0.0 | 0.0 | 7.0 |  | 1 | 1.9 | 0.0 | 9.9 |  | 0 | 0.0 | 0.0 | 7.4 |
|  | Lobar pneumonia (10024738) | 0 | 0.0 | 0.0 | 7.0 |  | 1 | 1.9 | 0.0 | 9.9 |  | 1 | 2.1 | 0.1 | 11.1 |
|  | Malaria (10025487) | 2 | 3.9 | 0.5 | 13.5 |  | 1 | 1.9 | 0.0 | 9.9 |  | 3 | 6.3 | 1.3 | 17.2 |
|  | Oral candidiasis (10030963) | 1 | 2.0 | 0.0 | 10.4 |  | 1 | 1.9 | 0.0 | 9.9 |  | 0 | 0.0 | 0.0 | 7.4 |
|  | Otitis media (10033078) | 0 | 0.0 | 0.0 | 7.0 |  | 0 | 0.0 | 0.0 | 6.6 |  | 2 | 4.2 | 0.5 | 14.3 |
|  | Pneumocystis jirovecii pneumonia (10073755) | 3 | 5.9 | 1.2 | 16.2 |  | 1 | 1.9 | 0.0 | 9.9 |  | 0 | 0.0 | 0.0 | 7.4 |
|  | Pneumonia (10035664) | 5 | 9.8 | 3.3 | 21.4 |  | 4 | 7.4 | 2.1 | 17.9 |  | 5 | 10.4 | 3.5 | 22.7 |
|  | Pulmonary tuberculosis (10037440) | 1 | 2.0 | 0.0 | 10.4 |  | 1 | 1.9 | 0.0 | 9.9 |  | 0 | 0.0 | 0.0 | 7.4 |
|  | Pyoderma (10037632) | 0 | 0.0 | 0.0 | 7.0 |  | 0 | 0.0 | 0.0 | 6.6 |  | 1 | 2.1 | 0.1 | 11.1 |
|  | Salmonella sepsis (10058878) | 0 | 0.0 | 0.0 | 7.0 |  | 1 | 1.9 | 0.0 | 9.9 |  | 0 | 0.0 | 0.0 | 7.4 |
|  | Sepsis (10040047) | 1 | 2.0 | 0.0 | 10.4 |  | 0 | 0.0 | 0.0 | 6.6 |  | 0 | 0.0 | 0.0 | 7.4 |
|  | Skin bacterial infection (10052891) | 1 | 2.0 | 0.0 | 10.4 |  | 0 | 0.0 | 0.0 | 6.6 |  | 0 | 0.0 | 0.0 | 7.4 |
|  | Typhoid fever (10045275) | 0 | 0.0 | 0.0 | 7.0 |  | 0 | 0.0 | 0.0 | 6.6 |  | 1 | 2.1 | 0.1 | 11.1 |
|  | Upper respiratory tract infection (10046306) | 2 | 3.9 | 0.5 | 13.5 |  | 2 | 3.7 | 0.5 | 12.7 |  | 1 | 2.1 | 0.1 | 11.1 |
| Metabolism and nutrition disorders (10027433) | Malnutrition (10061273) | 0 | 0.0 | 0.0 | 7.0 |  | 3 | 5.6 | 1.2 | 15.4 |  | 0 | 0.0 | 0.0 | 7.4 |
| Musculoskeletal and connective tissue disorders (10028395) | Arthritis (10003246) | 1 | 2.0 | 0.0 | 10.4 |  | 0 | 0.0 | 0.0 | 6.6 |  | 0 | 0.0 | 0.0 | 7.4 |
| Nervous system disorders (10029205) | Febrile convulsion (10016284) | 1 | 2.0 | 0.0 | 10.4 |  | 0 | 0.0 | 0.0 | 6.6 |  | 1 | 2.1 | 0.1 | 11.1 |
| Skin and subcutaneous tissue disorders (10040785) | Dermatitis (10012431) | 0 | 0.0 | 0.0 | 7.0 |  | 0 | 0.0 | 0.0 | 6.6 |  | 1 | 2.1 | 0.1 | 11.1 |

ITT, intent-to-treat; R3R, group receiving 4 doses of RTS,S/AS01; R3C, group receiving 3 doses of RTS,S/AS01 plus 1 dose of comparator vaccine; C3C, group receiving 4 doses of comparator vaccine; N, number of participants with at least one administered vaccine dose; n (%), number (percentage) of participants reporting the event at least once; CI, confidence interval; LL, lower limit; UL, upper limit; SAE, serious adverse event.

^a^While all children included in these analyses were confirmed to be HIV-infected, not all HIV infections were reported as SAEs; the decision to report an HIV infection as an SAE was based on the investigators’ judgment of whether it met the criteria of an SAE.

**Supplementary table 9. Number and percentage of HIV-infected participants for whom serious adverse events were reported during the 30-day period after dose 4, listed by Medical Dictionary for Regulatory Activities system organ class and preferred term (ITT population of HIV-infected participants)**

|  | | **R3R  N = 33** | | | |  | **R3C  N = 35** | | | | | |  | | **C3C  N = 28** | | | | | | |
| --- | --- | --- | --- | --- | --- | --- | --- | --- | --- | --- | --- | --- | --- | --- | --- | --- | --- | --- | --- | --- | --- |
|  | |  | | **95% CI** | |  |  | | | **95% CI** | | |  | |  | | | **95% CI** | | |  |
| **Primary System Organ Class (CODE)** | **Preferred Term (CODE)** | **n** | **%** | **LL** | **UL** |  | **n** | **%** | **LL** | | **UL** |  | | **n** | | **%** | **LL** | | **UL** |  |  |
| At least one SAE |  | 4 | 12.1 | 3.4 | 28.2 |  | 3 | 8.6 | 1.8 | | 23.1 |  | | 1 | | 3.6 | 0.1 | | 18.3 |  |  |
| Blood and lymphatic system disorders (10005329) | Anemia (10002034) | 1 | 3.0 | 0.1 | 15.8 |  | 0 | 0.0 | 0.0 | | 10.0 |  | | 0 | | 0.0 | 0.0 | | 12.3 |  |  |
| Infections and infestations (10021881) | Gastroenteritis (10017888) | 0 | 0.0 | 0.0 | 10.6 |  | 2 | 5.7 | 0.7 | | 19.2 |  | | 0 | | 0.0 | 0.0 | | 12.3 |  |  |
|  | HIV infection (10020161)^a^ | 1 | 3.0 | 0.1 | 15.8 |  | 1 | 2.9 | 0.1 | | 14.9 |  | | 0 | | 0.0 | 0.0 | | 12.3 |  |  |
|  | Malaria (10025487) | 1 | 3.0 | 0.1 | 15.8 |  | 1 | 2.9 | 0.1 | | 14.9 |  | | 0 | | 0.0 | 0.0 | | 12.3 |  |  |
|  | Pneumonia (10035664) | 1 | 3.0 | 0.1 | 15.8 |  | 0 | 0.0 | 0.0 | | 10.0 |  | | 1 | | 3.6 | 0.1 | | 18.3 |  |  |
|  | Salmonella sepsis (10058878) | 0 | 0.0 | 0.0 | 10.6 |  | 0 | 0.0 | 0.0 | | 10.0 |  | | 1 | | 3.6 | 0.1 | | 18.3 |  |  |
|  | Tuberculosis (10044755) | 1 | 3.0 | 0.1 | 15.8 |  | 0 | 0.0 | 0.0 | | 10.0 |  | | 0 | | 0.0 | 0.0 | | 12.3 |  |  |
| Metabolism and nutrition disorders (10027433) | Malnutrition (10061273) | 0 | 0.0 | 0.0 | 10.6 |  | 0 | 0.0 | 0.0 | | 10.0 |  | | 1 | | 3.6 | 0.1 | | 18.3 |  |  |
| Nervous system disorders (10029205) | Convulsion (10010904) | 0 | 0.0 | 0.0 | 10.6 |  | 1 | 2.9 | 0.1 | | 14.9 |  | | 0 | | 0.0 | 0.0 | | 12.3 |  |  |
|  | Febrile convulsion (10016284) | 1 | 3.0 | 0.1 | 15.8 |  | 0 | 0.0 | 0.0 | | 10.0 |  | | 0 | | 0.0 | 0.0 | | 12.3 |  |  |

ITT, intent-to-treat; R3R, group receiving 4 doses of RTS,S/AS01; R3C, group receiving 3 doses of RTS,S/AS01 plus 1 dose of comparator vaccine; C3C, group receiving 4 doses of comparator vaccine; N, number of participants who received the fourth vaccine dose; n (%), number (percentage) of participants reporting the event at least once; CI, confidence interval; LL, lower limit; UL, upper limit; SAE, serious adverse event.

^a^While all children included in these analyses were confirmed to be HIV-infected, not all HIV infections were reported as SAEs; the decision to report an HIV infection as an SAE was based on the investigators’ judgment of whether it met the criteria of an SAE.

**Supplementary figure 1. Distribution of age at positive HIV test in relation to age at enrollment (ITT population of HIV-infected participants)**

**R3R**

**R3C**

**C3C**

ITT, intent-to-treat; R3R, group receiving 4 doses of RTS,S/AS01 ; R3C, group receiving 3 doses of RTS,S/AS01 plus 1 dose of comparator vaccine; C3C, group receiving 4 doses of comparator vaccine.

**Supplementary figure 2. Survival curves by age category (ITT population of HIV-infected participants)**

**A. 5–17 months age category**

**
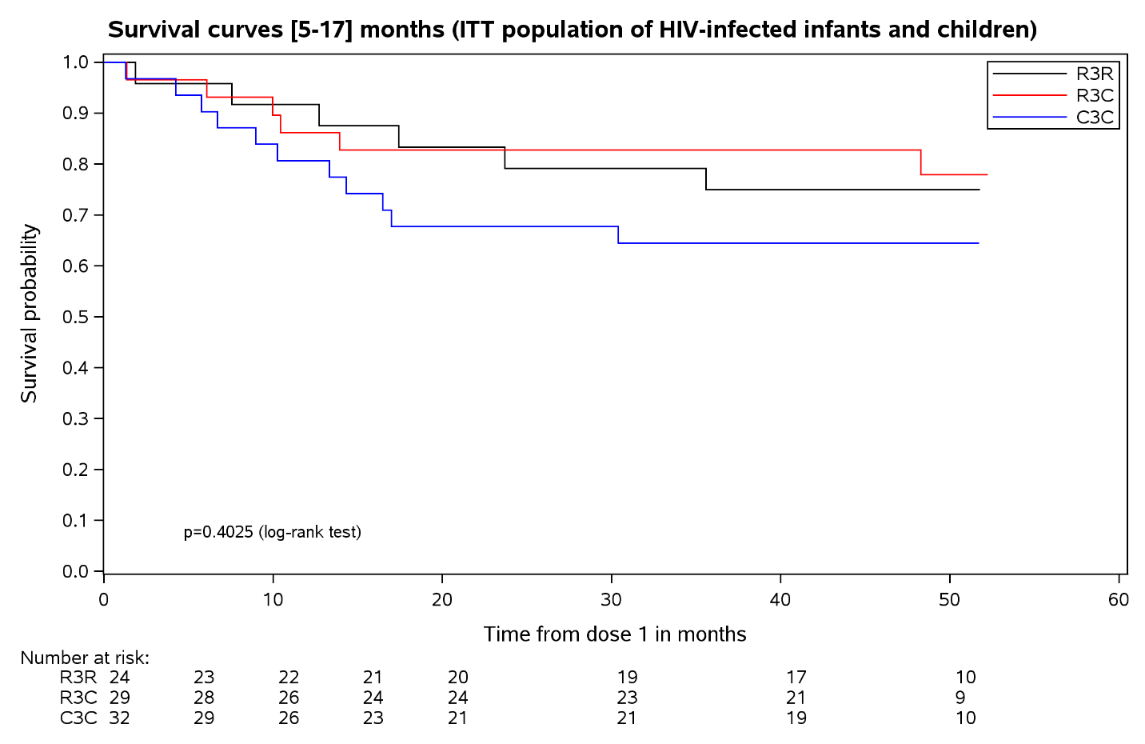
**

**B. 6–12 weeks age category**

**
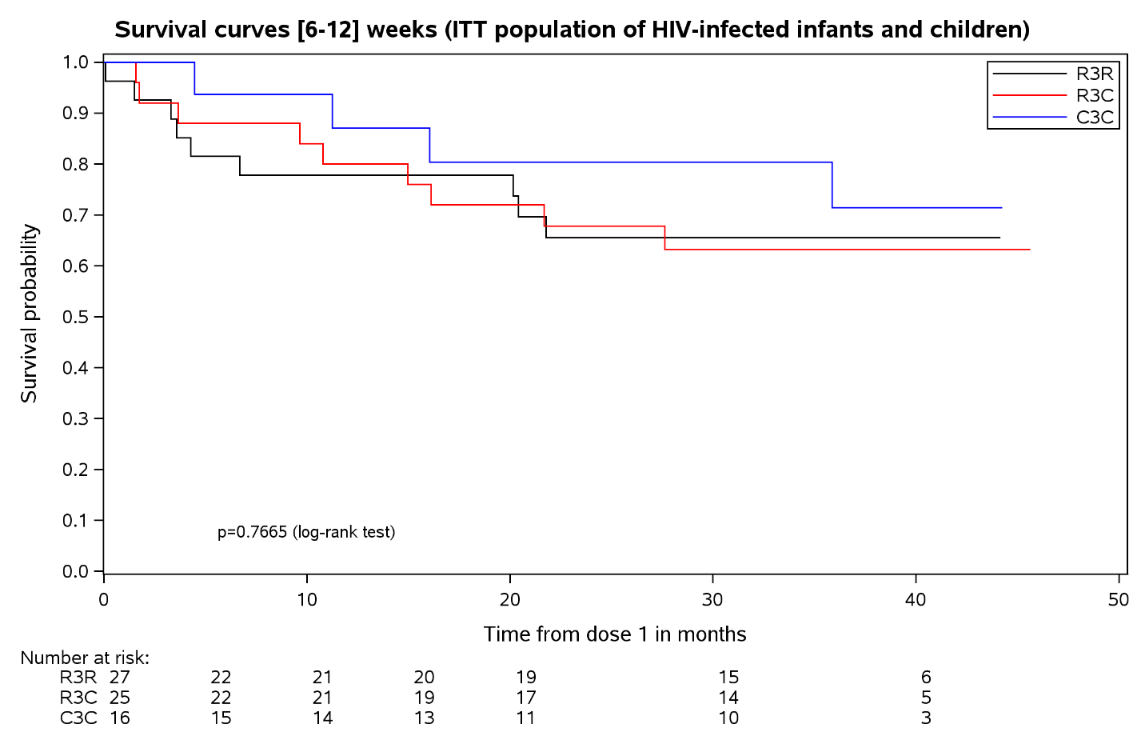
**

ITT, intent-to-treat; R3R, group receiving 4 doses of RTS,S/AS01 ; R3C, group receiving 3 doses of RTS,S/AS01 plus 1 dose of comparator vaccine; C3C, group receiving 4 doses of comparator vaccine.
